# New Schiff base ligand and its novel Cr(III), Mn(II), Co(II), Ni(II), Cu(II), Zn(II) complexes: spectral investigation, biological applications, and semiconducting properties

**DOI:** 10.1038/s41598-022-22713-z

**Published:** 2022-10-26

**Authors:** Mosad A. El-ghamry, Fatma M. Elzawawi, Ayman A. Abdel Aziz, Khadija M. Nassir, Samy M. Abu-El-Wafa

**Affiliations:** 1 Department of Chemistry, Faculty of Education, Ain Shams University, Roxy, 11711 Cairo Egypt; 2 Department of Chemistry, Faculty of Science, Ain Shams University, Abbasia, Cairo, Egypt

**Keywords:** Biochemistry, Computational biology and bioinformatics, Drug discovery, Chemistry, Nanoscience and technology

## Abstract

New Schiff base ligand, derived from antiviral valacyclovir, and its novel Cr(III), Mn(II), Co(II), Ni(II), Cu(II), Zn(II) complexes have been synthesized. By using a variety of analytical and spectroscopic techniques, the type of bonding between the ligand and the metal ions in the recently formed complexes was clarified. The Schiff base ligand act as a bidentate and coordinated with the metal ions through the azomethine-N and the phenolic-O centers, in a mono-deprotonated form. Except for the Zn(II) complex, which displayed a tetrahedral geometry, all complexes displayed octahedral geometry. The TGA findings supported that the stability and decomposition properties of the metal complexes were entirely distinct from one another. The thermogram showed decomposition of all investigated metal complexes above 200 °C in three, four or five steps, and indicated the high thermal stability of these complexes. According to XRD patterns, the particles of these complexes were located at the nanoscale. Moreover, for all the samples analyzed, the TEM images showed uniform and homogeneous surface morphology. The biological activity revealing the high efficiencies of the screened complexes as antibacterial and antitumor agents. The antimicrobial activity of the ligand and its complexes was examined against a variety of pathogenic bacteria and fungi including *Escherichia coli*, *Staphylococcus aureus* and *Candida albicans*. The data obtained revealed that the metal ion in the complexes enhanced the antimicrobial activity compared to the free ligand. The high efficiencies toward *S. aureus*, *E. coli*, and C. albicans appeared by Cu(II) complex 23, Ni(II) complex 20, and Ni(II) complex 19, respectively. The antitumor activity of the ligand and its complexes was tested against Hepatocellular carcinoma cell line (HepG-2 cells), the residue 28 which produced after heating the Cu(II) complex 25 at 200 °C for 1 h, exhibited strong inhibition of HepG-2 cell growth. The results of the DNA cleavage investigation demonstrated the ability of investigated Cu(II) complex to degrade DNA. The docking findings showed strong interactions of both the ligand and its examined Cu(II) complex, revealing their ability to cleavage DNA and their potent inhibitory effects on tumor cells. The electrical conductivity study confirmed that the ligand and its investigated complexes had semiconducting properties.

## Introduction

Valacyclovir is the l-valine ester of acyclovir. It is classified as a nucleoside analog DNA polymerase enzyme inhibitor. These analogues are structurally identical to the nucleosides that are composed of DNA. In the viral DNA, they are inserted, thereby terminating the growing viral DNA chain^1^. Valacyclovir has a high binding affinity for DNA-CT and its inhibitory function is highly selective because of its affinity for the thymidine kinase enzyme^2–4^. Valacyclovir shows varying levels of inhibition towards herpes simplex virus types 1 (HSV-1), 2 (HSV-2), Varicella Zoster Virus (VZV), Epstein-Barr virus (EBV), and cytomegalovirus (CMV). Moreover, Valacyclovir is the most effective antiviral medication used to treat FHV1 feline herpes virus infections^5–7^.

Recently, complexation has often been used to influence biological processes that are metal dependent^8,9^. The application of diagnostic metal complexes to medicine is a rapidly developing therapeutic field^10^. Cu(II) complex of Valacyclovir (VAL) with a formula [Cu(VAL)Cl_2_] has a cytotoxic effect against bovine herpes virus type I (BHV-1)^11^. Moreover, a binuclear Cu(II) complex of Valacyclovir (VAL) with a formula [Cu_2_(VAL)_3_(H_2_O)_4_] has high antifungal activity against the yeast cultures^12^.

Schiff bases and their complexes have variety of applications in biological and physiological activities including antimycobacterial, antifungal, anticonvulsant, antiallergenic, antiviral, antihypertensive and anticancer^13,14^. The green synthesis of nano coordination particles, using the natural ingredients present in plant extracts, offers an alternative, efficient, inexpensive, and environmentally friendly method to produce well-defined geometries of nanoparticles^15^.

In this study, we focus on the preparation of a new Schiff base ligand of valacyclovir and 2,3-dihydroxybenzaldehyd, and its novel Cr(III), Mn(II), Co(II), Ni(II), Cu(II), Zn(II) complexes. Numerous methods, including elemental analysis, FTIR, ESR, UV–Vis, 1HNMR, and mass spectra in addition to thermal and magnetic studies, were used to characterize the produced Schiff base and its complexes. Transmission electron microscopy (TEM) and powder X-ray diffraction (XRD) were used to determine the particle size and morphology of the complexes. The antimicrobial and antitumor capabilities of the newly synthesized compounds were investigated. The AC electrical conductivity in solid state at various temperatures for the Schiff base ligand and its complexes were tested. Computational studies including DFT, and molecular docking have been carried out. Additionally, DNA cleavage for the ligand and its complexes was examined.

## Experimental

### Materials

All materials used were provided by Sigma or Aldrich. They included valacyclovir (VACV), 2,3-dihydroxybenzaldehyd, LiOH·H_2_O, cetyltrimethylammonium bromide (CTAB), EDTA, CrCl_3_·6H_2_O, MnCl_2_·4H_2_O, CoCl_2_·6H_2_O, NiCl_2_·6H_2_O, CuCl_2_·2H_2_O, ZnCl_2_, and nitric acid. The solvents used, ethanol, methanol, dimethylformamide (DMF) and diethyl ether, were either spectroscopically pure or had undergone prescribed purification processes^16^ and tested for their spectral purity.

### Instrumentation and methods

The instruments that were employed in this study were those that previously discussed elsewhere^17^. The size and morphology of the complexes were examined under a transmission electron microscope (TEM) (JEOL 1010 Japan) using a standard protocol after loading on carbon-coated copper grides. The antimicrobial activities were screened against Gram positive (*Staphylococcus aureus*, ATCC 25923) and Gram negative (*Escherichia coli*, ATCC 25922) bacteria beside the unicellular fungus (*Candida albicans*, ATCC 10231) by applying the standardized disc—agar diffusion method^18,19^. Gaussian 09 W program was used to study the 3D-optimized molecular structures of the Schiff base ligand and its complexes by density functional theory (DFT) with Becke’s three-parameter exchange, and Lee–Yang–Parr correlation functional (B3LYP) with a combination of 6-311G++ (2d, p) basis set for the ligand, and LanL2DZ basis set for the complexes^20,21^. To evaluate the binding mode of the synthesized compounds on DNA, which is related to their activity, a molecular docking procedure was performed. Utilizing the Molecular Operating Environment (MOE, 2015.10) software^22^, all molecular modeling studies were carried out. All minimizations were carried out with MOE until an RMSD gradient of 0.05 kcal mol^−1^ Å^−1^ with MMFF94x force field and the partial charges were automatically determined. Gel electrophoresis experiment is used to perform DNA cleavage. Gel electrophoresis is a common technique for examining the interactions of compounds with nucleic acids: separation of molecules depending on their relative rate of movement through a gel when subjected to an electric field. A wide range of frequency, from 100 to 8 × 10^6^ Hz, and temperature, from 308 to 418 K, were used to study the AC conductivity (σ_ac_) of Schiff base ligand and its complexes.

### In vitro antitumor activity

Antitumor activities of the investigated compounds were recorded towards Hepatocellular carcinoma cell line, HepG-2 cells (ATCC No. HB-8064), on microplate reader (Sunrise, Tecan, Inc, USA) using 490 nm filters, and compared with the standard drug cis-platin (cis-diamminedichloroplatinium). The cell lines were purchased from American Type Culture Collection (ATCC; Rockville, MD, USA). The experiments were performed in the tissue culture unit at Regional Center for Mycology and Biotechnology, Al-Azhar University, Cairo, Egypt. Graphed prism software^23,24^ (San Diego, CA.USA) was used to calculate the 50% inhibitory concentration (IC_50_).

### Synthesis of the Schiff base ligand, DBAPB

**Figure 1:**
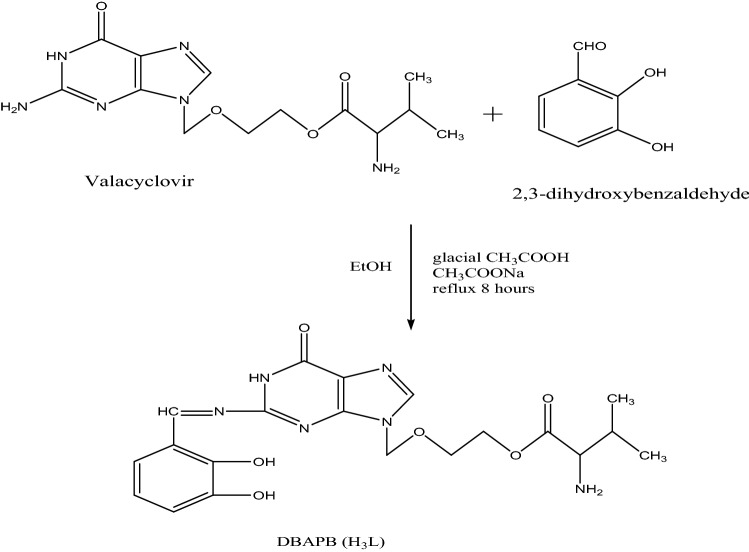
Synthesis of the Schiff base ligand, DBAPB.

**Figure 2:**
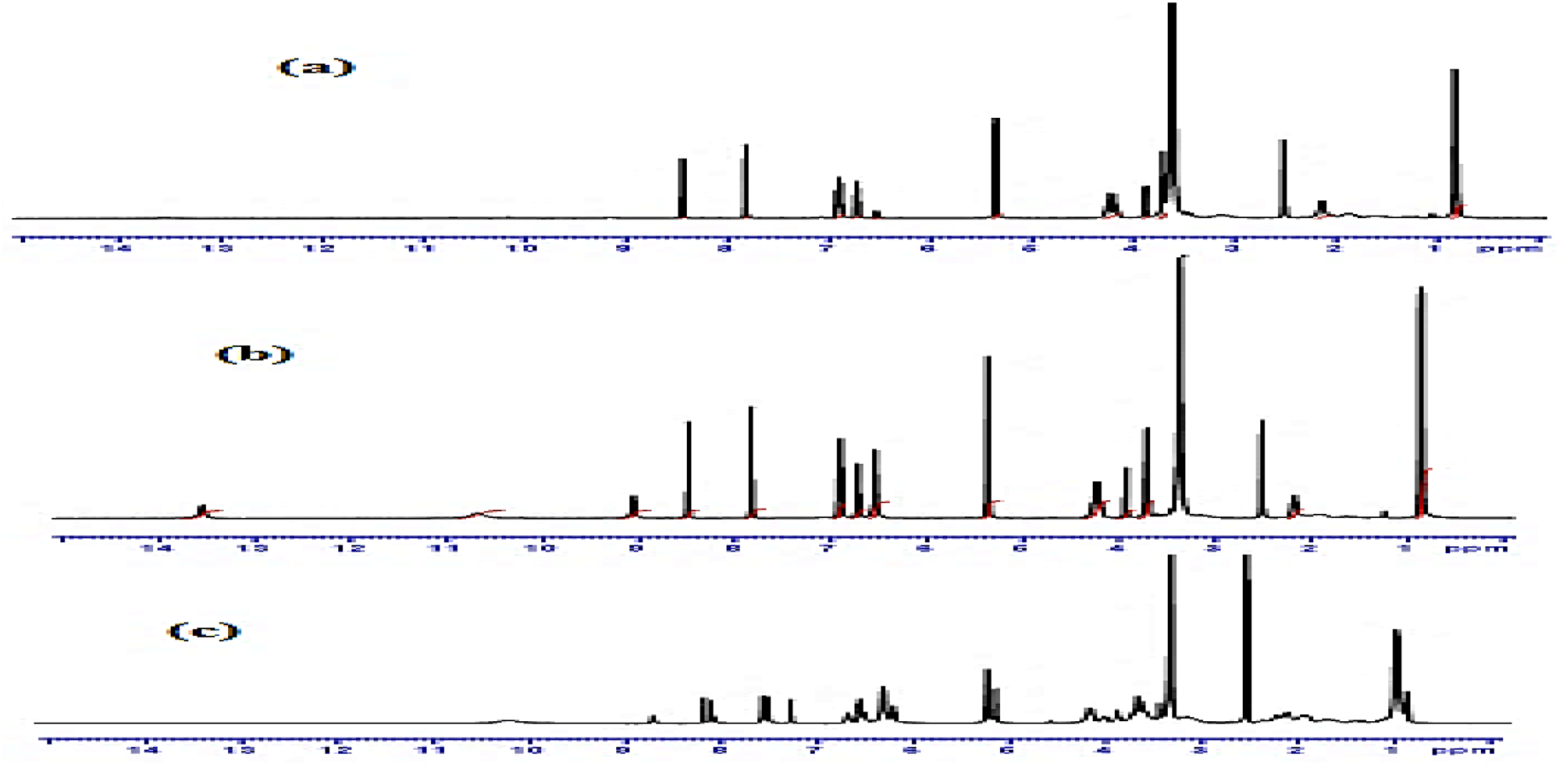
The ^1^HNMR spectra of the ligand, DBAPB, with D_2_O (**a**), without D_2_O (**b**), and Zn(II) complex 6 (**c**).

The Schiff base ligand, 2-{(2-[(2,3-dihydroxy-benzylidene)-amino]-6-oxo-1H-purine-9-yl)methoxy}ethyl-2-amino-3-mehyl butanoate, DBAPB was synthesized using the suggested procedure as previously reported elsewhere^25^, Fig. 1. After being allowed to cool to room temperature, the product was filtered out, recrystallized from ethanol, and then dried under vacuum to give yellow crystals, with a yield of 70% and a melting point of 170 °C. Evidence for the suggested structure of the ligand, DBAPB, is provided by ^1^HNMR spectra. Figure 2a,b illustrates the ^1^HNMR spectral data (δ ppm) of the ligand in DMSO-d6 with and without D_2_O. The data revealed signals at 0.84–0.86 [d, 6H, 2CH_3_], 2.21 [m, 1H, –CH(CH_3_)_2_], 3.73 [t, 2H, –OCH_2_], 3.93 [d, 1H, –CH–NH_2_], 4.28 [t, 2H, CH_2_OCO], 5.36 [s, 2H, NCH_2_O], 6.54 [s, 2H, –NH_2_], 6.70–6.92 [m, 3H, Ar–H], 7.83 [s, 1H, Ar–H 5-membered ring], 8.50 [s, 1H, CH=N], 9.04 [s, 1H, NH (disappeared on adding D_2_O)], 10.67 [s, 1H, OH (disappeared on adding D_2_O)], 13.54 [s, 1H, OH (completely disappeared on adding D_2_O)^26^.

### Synthesis of the metal complexes

Novel metal complexes of the Schiff base ligand, DBAPB, have been prepared in EtOH in a 1:1 (M:L) molar ratio, in addition of CTAB (3 × 10^–2^ M), Malva parviflora extract (MP) (20%), or Spinacia oleracea extract (SO) (20%) in EtOH. [where, M = Cr(III), Mn(II), Co(II), Ni(II), Cu(II) or Zn(II)] By using the experimental procedures that previously reported^27^. The solid complexes obtained (in EtOH, CTAB/EtOH, SO/EtOH, MP/EtOH): Cr(III) complexes **1**, **7**, **8**, **9**, Mn(II) complexes **2**, **11**, **12**, **13**, Co(II) complexes **3**, **15**, **16**, **17**, Ni(II) complexes **4**, **19**, **20**, **21**, Cu(II) complexes **5**, **23**, **24**, **25** and Zn(II) complexes **6**, **29**, **30**, **31** were filtered off, ethanol-washed and finally vacuum-dried over anhydrous CaCl_2_. Table 1 contains the analytical and physical data for the metal complexes.

**Table 1:** Physicochemical properties of the metal complexes of the Schiff base ligand, DBAPB.

No	Complex	Molecular formula	Color	%Yield	M.Wt (gm mol^−1^)	Elemental analysis, found % (calcd %)				Ω_m_ (ohm^−1^ cm^2^ mol^−1^)
						% C	% H	% N	% M	
	DBAPB	C_20_H_24_N_6_O_6_	Yellow	70	444.44	53.24 (54.05)	5.10 (5.41)	18.81 (18.92)	–	–
1	[Cr(DBAPB)(H_2_O)_2_Cl_2_]	C_20_H_27_N_6_O_8_Cl_2_Cr	Olive green	77	601.9	39.94 (39.87)	5.00 (4.49)	13.75 (13.96)	8.00 (8.62)	5.0
2	[Mn(DBAPB)(EtOH)_3_Cl]	C_26_H_41_N_6_O_9_ClMn	Brown	75	671.4	46.31 (46.47)	6.58 (6.11)	13.08 (12.51)	9.29 (8.18)	5.0
3	[Co(DBAPB)(EtOH)_2_(H_2_O)Cl].H_2_O	C_24_H_39_N_6_O_10_ClCo	Brown	74	665.4	43.58 (43.28)	5.00 (5.86)	12.45 (12.62)	8.06 (8.85)	4.0
4	[Ni(DBAPB)(EtOH)_3_Cl].EtOH	C_28_H_47_N_6_O_10_ClNi	Olive green	62	721.1	47.39 (46.59)	6.97 (6.52)	11.27 (11.65)	8.41 (8.13)	7.0
5	[Cu(DBAPB)(H_2_O)_3_Cl]	C_20_H_29_N_6_O_9_ClCu	Green	67	596.0	40.65 (40.27)	4.56 (4.87)	14.42 (14.09)	10.72 (10.65)	11. 0
6	[Zn(DBAPB)(EtOH)Cl]	C_22_H_29_N_6_O_7_ClZn	yellow	73	589.8	45.44 (44.76)	5.56 (4.92)	14.50 (14.24)	10.97 (11.07)	6.0

## Results and discussion

### Characterization of the Schiff base ligand, DBAPB

**Figure 3:**
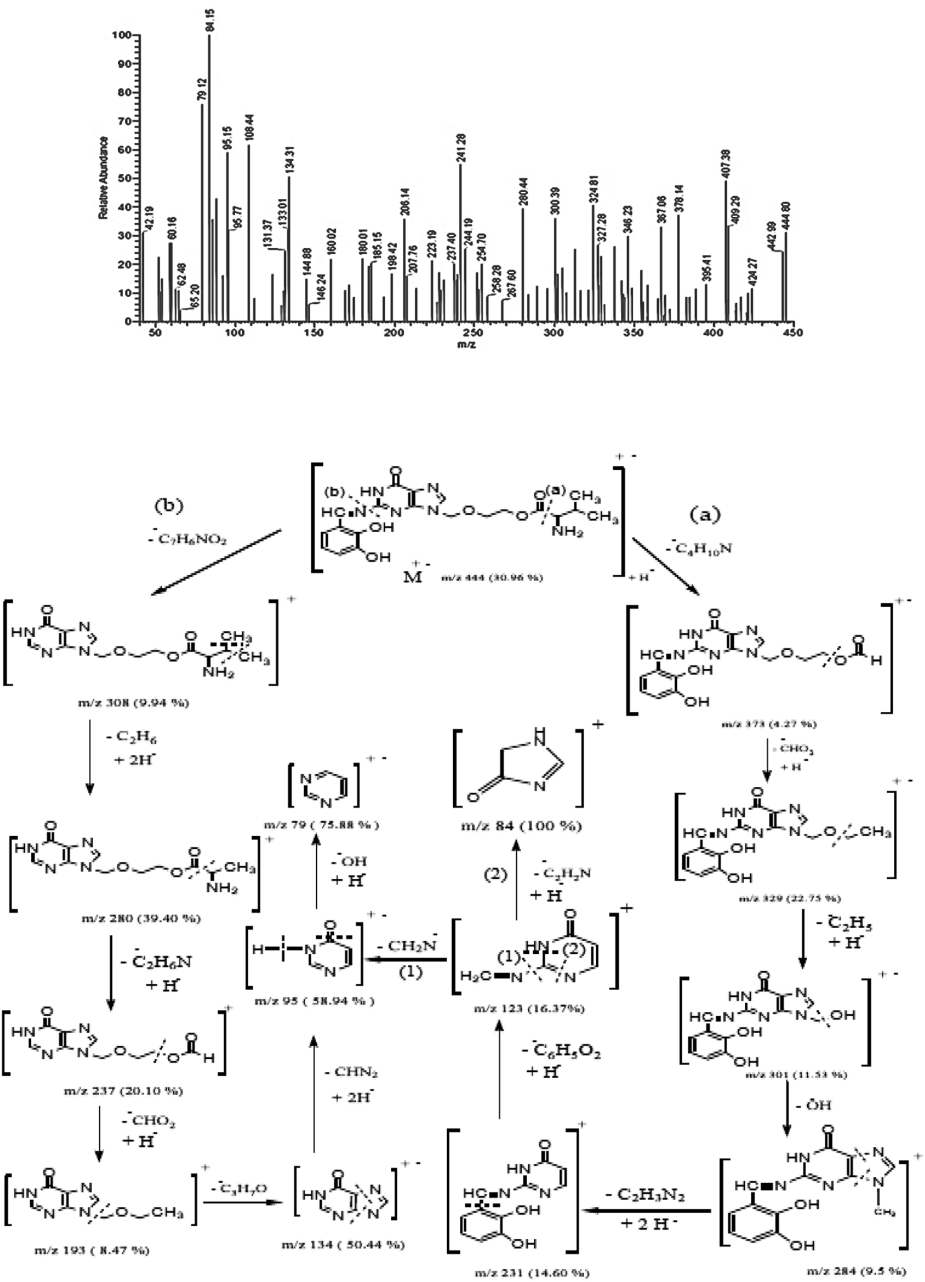
Mass spectrum and mass fragmentation of the ligand, DBAPB.

The results of elemental analysis, IR, ^1^HNMR, UV–Vis, and mass spectra studies confirmed the purity of the Schiff base ligand, DBAPB. The findings confirmed the percentage of net chemical composing. Anal. Calcd % C, 54.05; H, 5.41; N, 18.92. Found %: C, 53.24; H, 5.10; N, 18.81. The formula weight (F.W. = 444.44) was confirmed by the molecular ion peak at m/z = 444.80 amu in the mass spectrum of the Schiff base ligand, DBAPB, Fig. 3. The mass fragmentation pattern, Fig. 3, supported the DBAPB 's proposed structure. The typical bands for υ(OH) phenolic at 3453, 3413, υ(NH) at 3191, υ(C=O)ester at 1728, υ(C=O)amide at 1690, δ(NH) at 1632, υ(C=N)azomethine at 1603 cm^−1^^28^, were observed in the IR spectrum (cm^−1^) of the ligand, DBAPB, Table 2. The electronic spectrum, λ_max_ (nm), of the ligand (10^−3^ M in DMF) at room temperature showed three absorption bands at 293, 346, and 427 nm corresponded to the intramolecular charge transfer (CT) transitions within the entire molecule.

**Table 2:** IR spectral data (cm^−1^) of the Schiff base ligand, DBAPB, and its metal complexes.

No	Compound or complex	IR spectral bands (cm^−1^)										
		υ(OH) EtOH /H_2_O	υOH) phenolic	υ(NH)	υ(CH) aromatic	υ(CH) aliphatic	υ(C=O) ester	υ(C=O) amide	δ(NH)	υ(C=N) azomethine	υ(M–O)	υ(M–N)
	DBAPB (H_3_L)	–	3453, 3413 (m)	3191 (m)	3042 (w)	2963 (w)	1728 (s)	1690 (s)	1632 (m)	1603 (s)	–	–
1	[Cr(DBAPB)(H_2_O)_2_Cl_2_]	3455 (br)	–, 3419 (m)	3178 (m)	3048 (w)	2963 (w)	1742 (s)	1686 (s)	1641 (m)	1593 (s)	501 (w)	440 (w)
2	[Mn(DBAPB)(EtOH)_3_Cl]	3433 (br)	–, 3416 (m)	3169 (m)	3048 (w)	2961 (w)	1736 (s)	1680 (s)	1632 (m)	1595 (s)	503 (w)	429 (w)
3	[Co(DBAPB)(EtOH)_2_(H_2_O)Cl].H_2_O	3445 (br)	–, 3417 (m)	3172 (m)	3042 (w)	2963 (w)	1730 (s)	1679 (s)	1629 (m)	1586 (s)	507 (w)	437 (w)
4	[Ni(DBAPB)(EtOH)_3_Cl].EtOH	3445 (br)	–, 3418 (m)	3172 (m)	3042 (w)	2962 (w)	1737 (s)	1680 (s)	1636 (m)	1595 (s)	507 (w)	417 (w)
5	[Cu(DBAPB)(H_2_O)_3_Cl]	3439 (br)	–, 3415 (m)	3172 (m)	3035 (w)	2963 (w)	1739 (s)	1686 (s)	1634 (m)	1589 (s)	501 (w)	459 (w)
6	[Zn(DBAPB)(EtOH)Cl]	3425 (br)	–, 3417 (m)	3194 (m)	3035 (w)	2962 (w)	1731 (s)	1686 (s)	1631 (m)	1589 (s)	501 (w)	457 (w)

*br* broad, *m* medium, *w* weak, *s* strong.

### Characterization of the metal complexes

The compositions and chemical formulae listed in Table 1 were ascribed to the metal complexes **1**–**6** of the ligand, DBAPB. The results of the elemental analysis showed that all complexes formed with a 1:1 (M:L) stoichiometry. The molar conductance values (Ω) of the complexes **1**–**6**, Table 1, in DMF (10^–3^ M) at room temperature ranged from 4.0 to 11.0 Ω^−1^cm^2^ mol^−1^. These values indicated that these complexes were non-electrolytes^29^ and that the chloride ions were contained within the coordination sphere.

### Infrared spectra

The IR spectrum of the ligand, DBAPB, was compared to those of the metal complexes. The characteristic infrared spectral bands of the free ligand and its metal complexes, together with their assignments, are listed in Table 2. All the complexes **1**–**6** showed a broad band in the 3425–3455 cm^−1^ range that was attributed to the υ(OH) of the H_2_O and/or EtOH molecules associated with the complexes. The IR spectrum of the free ligand revealed two distinctive bands at 3453 and 3413 cm^−1^ which are attributed to the OH– phenolic frequencies. One of the υ(OH) phenolic bands was disappeared in the IR spectra of all metal complexes and the other band was almost coincident with the corresponding one of the free ligand indicating that one of the phenolic OH groups is deprotonated by the coordination phase, while the other phenolic-OH group was not involved in complex formation. In the IR spectra of the metal complexes, the band corresponding to υ(C=N) which was present at 1603 cm^−1^ in the IR spectrum of the free ligand, was shifted to a lower wave number by 8–17 cm^−1^, indicating the presence of the azomethine group's nitrogen atom in bonding with the metal ion^30^. The distinctive vibrations of the purinone moiety's (C=O)amide and the aliphatic side chain's (C=O)ester were essentially unaltered by the complexation, indicating that these groups were not involved in coordination. According to the IR spectral data we can conclude that the ligand, DBAPB, act as a bidentate and coordinated with the metal ions through the azomethine-N and the phenolic-O centers, in a mono-deprotonated form. The newly formed bands at 501–507 and 417–459 cm^−1^ in the IR spectra of the complexes were attributed to the metal–oxygen and the metal-nitrogen vibrations, respectively^31^, and confirmed the metal–ligand bonding.

### ^1^HNMR spectra

In order to confirm the bonding mode between the ligand, DBAPB, and the Zn(II) ion. The ^1^HNMR spectrum of Zn(II) complex **6**, Fig. 2c, was recorded. The signals detected at δ 1.22, 2.41 and 3.63 ppm characteristic for the specific protons of coordinated EtOH in the Zn(II) complex. The singlet detected at δ 13.54 ppm due to the phenolic-OH proton in the free ligand, is not present in the Zn(II) complex, indicating that the phenolic-OH group was deprotonated through coordination with the metal ion. While the singlet (δ 10.67 ppm) for proton of the other phenolic-OH group appearing approximately in the same position as in the free ligand, suggesting the other phenolic-OH group was not involved in complex formation. A chelation of the azomethine-N atom with the metal ion is indicated by the shifting of the band of the azomethine proton (–CH=N–) from δ 8.50 ppm in the ^1^HNMR spectrum of free ligand to δ 8.40 ppm in the ^1^HNMR spectrum of Zn(II) complex **6**. These findings coincide with the conclusions drawn from IR spectral studies.

### Thermal analysis

Thermal analysis is used extensively to study the stability and decomposition characteristics of the metal complexes. The thermal behavior of the synthesized complexes **1**–**6** have been investigated to create various decomposition processes and to validate the proposed stoichiometry. TGA was performed in the nitrogen environment between room temperature and 900 °C. The results of the TGA investigation of complexes **1**–**6**, Table 3, showing a strong correlation with the weight loss values measured and found.

**Table 3:** Results of thermogravimetric analysis (TGA) of metal complexes of the ligand, DBABP.

Complex no	Molecular formula (M.wt)	Temperature range (°C)	Wt loss %Found % (calcd %)	Decomposed product lost
1	[Cr(DBABP)(H_2_O)_2_Cl_2_] (601.90)	22–214 215–321 322–659 660–887	5.40 (5.98) 19.31 (19.44) 32.54 (32.07) 24.58 (23.92) 18.17 (18.59)	2 coordinated H_2_O 2 HCl + CO_2_ CH_4_ + 2 C_2_H_2_ + CO_2_ + 3 HCN 0.5 HCHO + 0.5 CH_4_ + 3 HCN + C_3_H_4_ 0.5 Cr_2_O_3_ + 3 C
2	[Mn(DBABP)(EtOH)_3_Cl] (671.40)	42–162 163–297 298–884	6.80 (6.85) 19.13 (19.14) 53.50 (52.73) 20.57 (21.28)	1 coordinated EtOH 2 coordinated EtOH + HCl 6 HCN + 4 CH_4_ + CO_2_ + 3 CO MnO + 6 C
3	[Co(DBABP)(EtOH)_2_(H_2_O)Cl].H_2_O (665.40)	37–137 138–320 321–360 361–487 488–899	5.93 (5.41) 13.30 (13.83) 5.47 (5.49) 19.75 (19.83) 18.61 (18.94) 36.94 (36.50)	1 lattice H_2_O + 1 coordinated H_2_O 2 coordinated EtOH HCl 2 CO_2_ + CO + CH_4_ C_2_H_6_ + 3 N_2_H_4_ CoO + 14 C
4	[Ni(DBABP)(EtOH)_3_Cl].EtOH (721.10)	24–285 286–544 545–880	25.19 (25.52) 24.64 (24.06) 30.57 (30.09) 19.60 (20.33)	1 lattice EtOH + 3 coordinated EtOH HCl + 3 HCN + 2 CO 3 HCN + CO_2_ + CO + 4 CH_4_ NiO + 6 C
5	[Cu(DBABP)(H_2_O)_3_Cl] (596.00)	39–249 250–343 344–527 528–724 725–902	9.83 (9.06) 13.56 (13.51) 24.74 (24.50) 15.51 (16.28) 12.55 (13.25) 23.89 (23.40)	3 coordinated H_2_O CO_2_ + HCl CH_4_ + CO_2_ + N_2_H_4_ + 2 HCN CO + HCN + C_2_H_2_ + CH_4_ 2 C_2_H_2_ + HCN CuO + 5 C
6	[Zn(DBABP)(EtOH)Cl] (589.80)	38–243 244–307 308–445 446–900	7.90 (7.80) 11.04 (10.94) 24.28 (24.08) 43.36 (43.40) 13.42 (13.78)	1 coordinated EtOH HCl + CO 2 HCN + 2 CO_2_ C_2_H_4_ + CH_4_ + 4 HCN + 4 C_2_H_2_ ZnO

**Figure 4:**
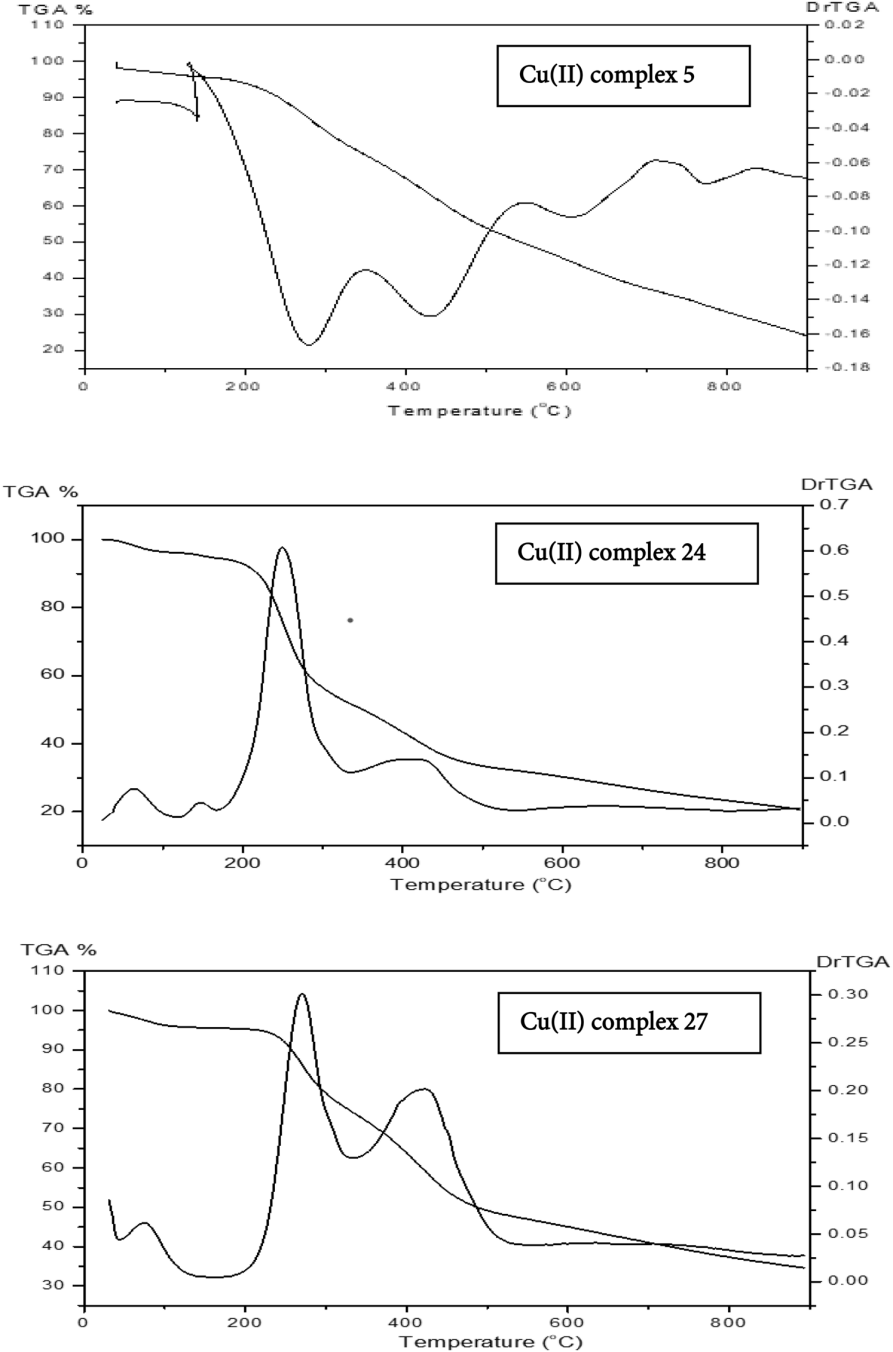
TGA–Dr TGA curves of the Cu(II) complexes 5, 24 and 27.

The novel complexes **1**–**6** prepared in EtOH, **8**, **12**, **16**, **20**, **24**, **30** prepared in SO/EtOH, and **10**, **14**, **18**, **22**, **27**, **32** produced after heating the corresponding complexes **8**, **12**, **16**, **20**, **24**, and **30**, respectively, at 200 °C for 1 h displayed different thermal decomposition behavior (thermal stabilities). The TG curves, Fig. 4, of the Cu(II) complexes **5**, **24** and **27** were described in detail as illustrative examples. Five decomposition steps were showed in the thermogram of complex **5** with midpoint temperatures of 137, 277, 428, 611 and 772 °C, respectively. Four decomposition stages were showed in the thermogram of complex **24** with midpoint temperatures of 61, 145, 250 and 422 °C, respectively. While the thermogram of complex **27** showed three decomposition steps at midpoint temperatures of 76, 267, and 422 °C, respectively, indicating their thermal stability in the following order: Complex **5** > Complex **27** > Complex **24**.

### Electronic spectra and magnetic moment measurements

The magnetic moments of the examined metal complexes as well as the electronic absorption spectra in DMF (10^–3^ M) were measured at room temperature. Table 4 displayed the electronic spectral data and the magnetic moment values (B.M.) of the Cr(III), Mn(II), Co(II), Ni(II) and Cu(II) complexes. The Cr(III) complex **1** displayed two absorption bands at 605 and 665 nm corresponded to the transitions ^4^A_2g_(F) → ^4^T_2g_(F) and ^4^A_2g_(F) → ^4^T_1g_(P), respectively, in an octahedral configuration. The octahedral geometry around the Cr(III) ion was confirmed by the Cr(III) complex's magnetic moment value of 3.35 B.M.^32^. One absorption band was exhibited in the electronic spectrum of Mn(II) complex **2** at 586 nm as a result of the ^6^A_1g_ → ^4^T_2g_(G) transition in an octahedral geometry. The octahedral geometry of the Mn(II) complex was confirmed by the Mn(II) complex's magnetic moment value of 5.22 B.M.^33^. Co(II) complex **3** displayed a distinctive band at 580 nm, which may be attributed to ^4^T_1g_(F) → ^4^A_2g_(F) transition in an octahedral configuration. The magnetic moment value = 4.42 B.M., which indicated the presence of three unpaired electrons in an octahedral arrangement^34^. Electronic spectrum of the Ni(II) complex **4** revealed two bands at 597 and 650 nm corresponded to the ^3^A_2g_(F) → ^3^T_1g_(F) and ^3^A_2g_(F) → ^3^T_1g_(P) transitions, respectively, in an octahedral stereochemistry. The magnetic moment value is found to be 3.19 B.M., indicating the octahedral geometry around the Ni(II) ion^35^. The electronic spectrum of Cu(II) complex **5** contains two bands at 605 and 673 nm attributed to ^2^B_1g_ → ^2^E_g_ and ^2^B_1g_ → ^2^B_2g_ transitions, respectively. The magnetic moment value = 2.14 B.M. which pointing to the presence of one unpaired electron in an octahedral configuration^36^. The magnetic moment value of the present Cu(II) complex was found to be 2.14 B.M.; which is quite high. This finding suggest spin–spin ferromagnetic coupling interaction, moments of the two unpaired electrons in the two adjacent Cu(II) ions are parallel to each other (↑ ↑), and the spins of the two electrons are in the same direction, which increases the magnetic moment value and become higher than the expected value.

**Table 4:** Electronic absorption spectral data (in 10^–3^ M DMF solution) and magnetic moment values of the metal complexes of the ligand, DBABP.

No	Complex	Color	λ_max_ (nm)	ε_max_ (mol^−1^ cm^−1^ L)	Assignment	Magnetic moment values (B.M.)
1	[Cr(DBABP)(H_2_O)_2_Cl_2_]	Olive green	605, 665	306.9, 463.8	^4^A_2g_(F) → ^4^T_2g_(F) ^4^A_2g_(F) → ^4^T_1g_(P)	3.35
2	[ Mn(DBABP)(EtOH)_3_Cl]	Brown	586	378.1	^6^A_1g_ → ^4^T_2g_(G)	5.22
3	[Co(DBABP)(EtOH)_2_(H_2_O)Cl].H_2_O	Brown	580	135.5	^4^T_1g_(F) → ^4^A_2g_(F)	4.42
4	[Ni(DBABP)(EtOH)_3_Cl].EtOH	Olive green	597, 650	116.9, 85.7	^3^A_2g_(F) → ^3^T_1g_(F) ^3^A_2g_(F) → ^3^T_1g_(P)	3.19
5	[Cu(DBABP)(H_2_O)_3_Cl]	Green	605, 673	264.9, 421.5	^2^B_1g_ → ^2^E_g_ ^2^B_1g_ → ^2^B_2g_	2.14

### ESR spectra

**Figure 5:**
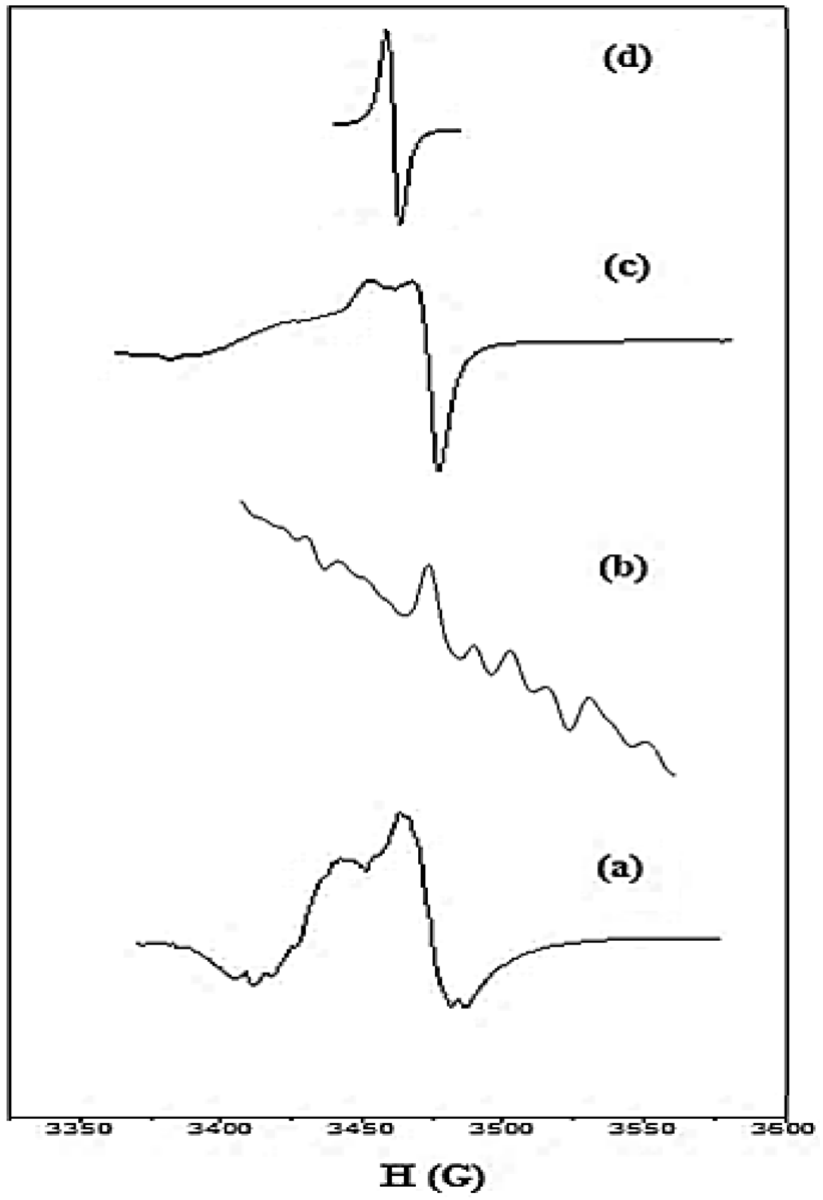
X-band ESR spectra of Mn(II) complex 2 (**a**), Co(II) complex 3 (**b**), Cu(II) complex 5 (**c**), and the standard (DPPH) (**d**).

The ESR spectra of powder Mn(II), Co(II), and Cu(II) complexes **2**, **3**, and **5**, respectively, were recorded at room temperature and represented in Fig. 5. The ESR spectrum of Mn(II) complex **2**, Fig. 5a, showed a single isotropic signal split into six hyperfine signals due to interaction with the nuclear spin of ^55^Mn^37^. The g_eff_ -value (2.1137) of Mn(II) complex is greater than that of a free electron (2.0023), indicating that the ligand and Mn(II) ion are bound together covalently. The ESR spectrum of Co(II) complex **3**, Fig. 5b, displayed one signal divided into 8 lines due to the hyperfine interaction with nuclear spin of ^59^Co. The g_eff_-value of Co(II) complex is 2.0129. The positive deviation from the free electron value (2.0023), suggesting the covalent character of the bond between the Co(II) ion and the ligand^38^. The X-band ESR spectrum of Cu(II) complex **5**, Fig. 5c, showed two signals with two different g-values, 2.113 and 2.059 for g_׀׀_ and g_⊥_, respectively, and g_av_ = 2.095. The shape of the ESR spectrum with the g-tensor values revealed that the examined Cu(II) complex **5** possesses octahedral geometry^39^. g_׀׀_ > g_⊥_ > 2.0023, suggesting the unpaired electron lies in the d_x_^2^_-y_^2^ orbital giving ^2^B_1g_ as the ground state^40^. The g_׀׀_ value < 2.3, indicating covalent nature for Cu-L bond^41^. The exchange interaction term (G) can be calculated using the formula G = (g_׀׀_ − 2.0023)/(g_⊥_ − 2.0023). The local tetragonal axes are parallel or just slightly misaligned if G > 4.0, while a substantial exchange coupling and misalignment is noticeable if G < 4.0^42^. The estimated G-value for the Cu(II) complex **5** was 1.94, indicating that the exchange coupling effects were operative between Cu(II) centers in the current Cu(II) complex^43^.

### Powder XRD studies

**Figure 6:**
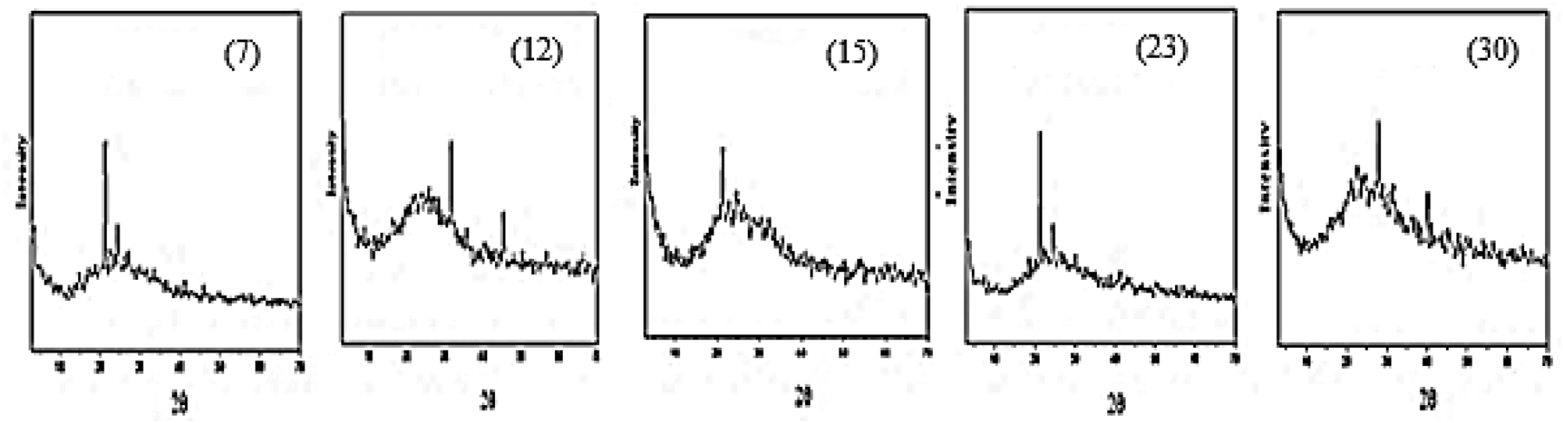
X-ray diffraction patterns of the nano Cr(III) 7, Mn(II) 12, Co(II) 15, Cu(II) 23 and Zn(II) 30 complexes.

X-ray diffraction of the Cr(III) complexes **7**, **8**, **9**, Mn(II) complexes **11**, **12**, **13**, Co(II) complexes **15**, **16**, **17**, Ni(II) complexes **19**, **20**, **21**, Cu(II) complexes **23**, **24**, **25** and Zn(II) complexes **29**, **30**, **31**, which prepared in (CTAB/EtOH, SO/EtOH, and MP/EtOH), respectively, have been studied, Fig. 6. According to X-ray diffractograms, the complexes **7**, **12**, **15**, **23**, and **30** have crystalline nature, whereas the complexes **8**, **9**, **11**, **13**, **16**, **17**, **19**, **20**, **21**, **24**, **25**, **29**, and **31** are amorphous. Debye Scherrer equation (d = Kλ/βCosθ) can be used to determine the particle size^44^. Where: D, K, λ, β, and θ are the particle size, the dimensionless shape factor, the X-ray wavelength, the full width at half maximum of the diffraction peak, and the diffraction angle, respectively. The crystalline nature complexes **7**, **12**, **15**, **23**, and **30** had average crystallite sizes of 21.10, 19.95, 3.00, 13.83 and 22.70 nm, respectively, indicating that the particles of these complexes were in nano scale.

### TEM studies

**Figure 7:**
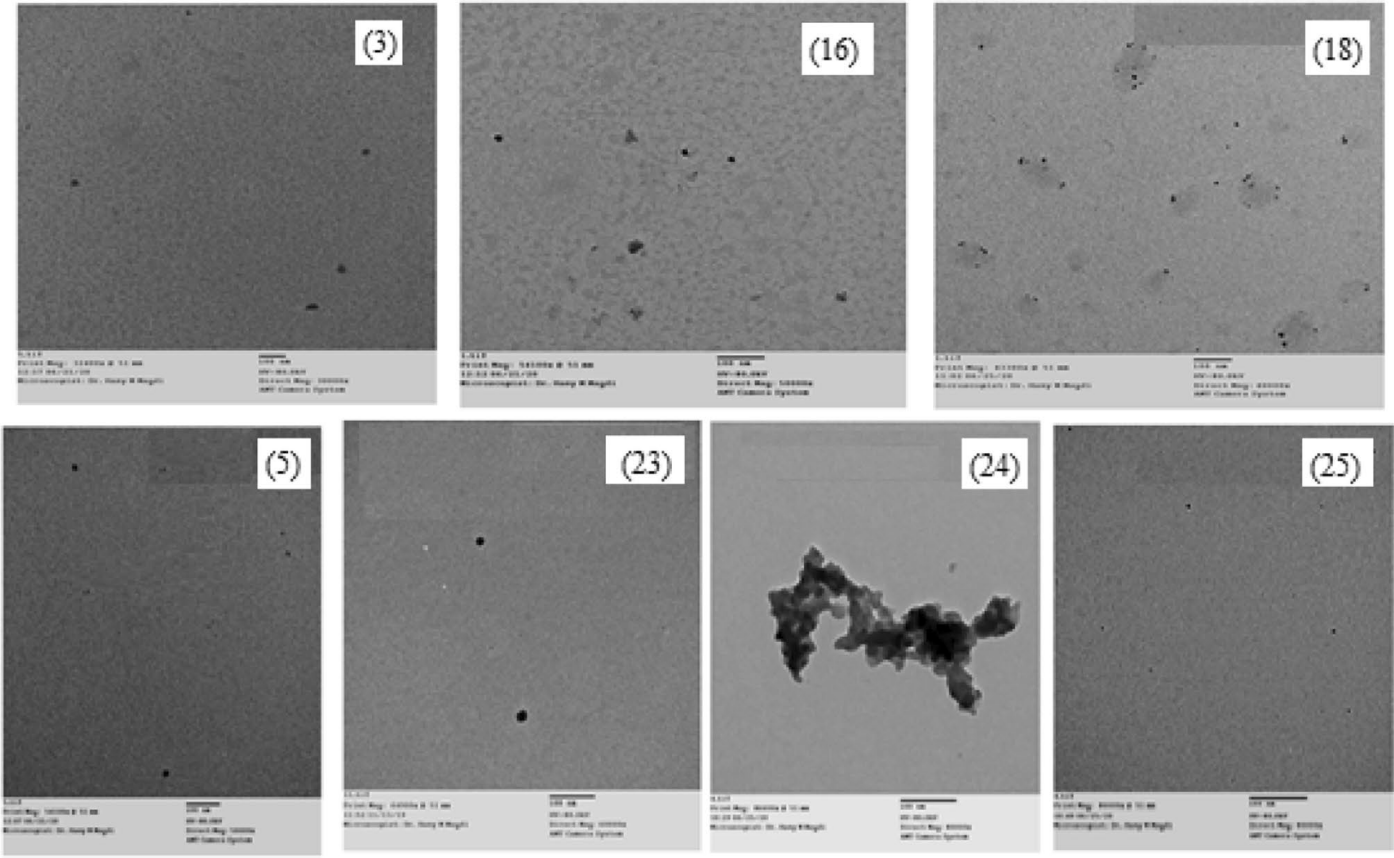
TME images of the nano Co(II) complexes 3, 16, 18 and the nano Cu(II) complexes 5, 23, 24, 25.

Transmission electron microscope (TEM) is widely used technique for detecting the particle size and form of solid materials, it is frequently used to reveal numerous nano metal complexes^45^. TEM analysis was conducted on Cr(III), Mn(II), Co(II), and Cu(II) complexes **1**, **2**, **3**, and **5** prepared in EtOH, **8**, **12**, **16**, and **24** prepared in SO/EtOH, **10**, **14**, **18**, and **27** produced after heating the corresponding complexes **8**, **12**, **16**, and **24**, respectively, at 200 °C for 1 h, as well as Cu(II) complexes **23** and **25** prepared in CTAB/EtOH and MP/EtOH, respectively, **26** and **28** produced after heating the corresponding complexes **23** and **25** respectively, at 200 °C for 1 h, to study the morphology and particle size of them. TEM images of the Co(II) complexes **3**, **16**, **18**, and Cu(II) complexes **5**, **23**, **24**, **25** were showed in Fig. 7. The micrographs represent distinct particle forms in nanocrystalline matrices. All the samples analyzed show a consistent and homogeneous surface morphology. The images show the uniformity and proximity of particle shapes, which supports the existence of identical matrices. The inclusion of strongly symmetrical spherical anions in the complexation sphere typically results in the existence of the spherical character. The particle sizes of complexes **1**, **2**, **3** and **5** were 11.87, 45.15, 28.6 and 18.06 nm, respectively. The particle size of complexes **8**, **12**, **16**, **24** decreased from 10.05, 16.90, 15.10 and 14.48 nm to 4.33, 3.22, 5.11 and 7.77 nm in their resulting residues **10**, **14**, **18** and **27**, respectively. Additionally, the TEM images, Figs. 10 and 11, demonstrated decrease in particle size of Cu(II) complexes **23** and **25** from 14.28 and 8.6 nm to 3.14 and 3.51 nm in their resulting residues **26** and **28**, respectively.

## Molecular modeling and DFT calculation studies

### Geometrical optimization

**Figure 8:**
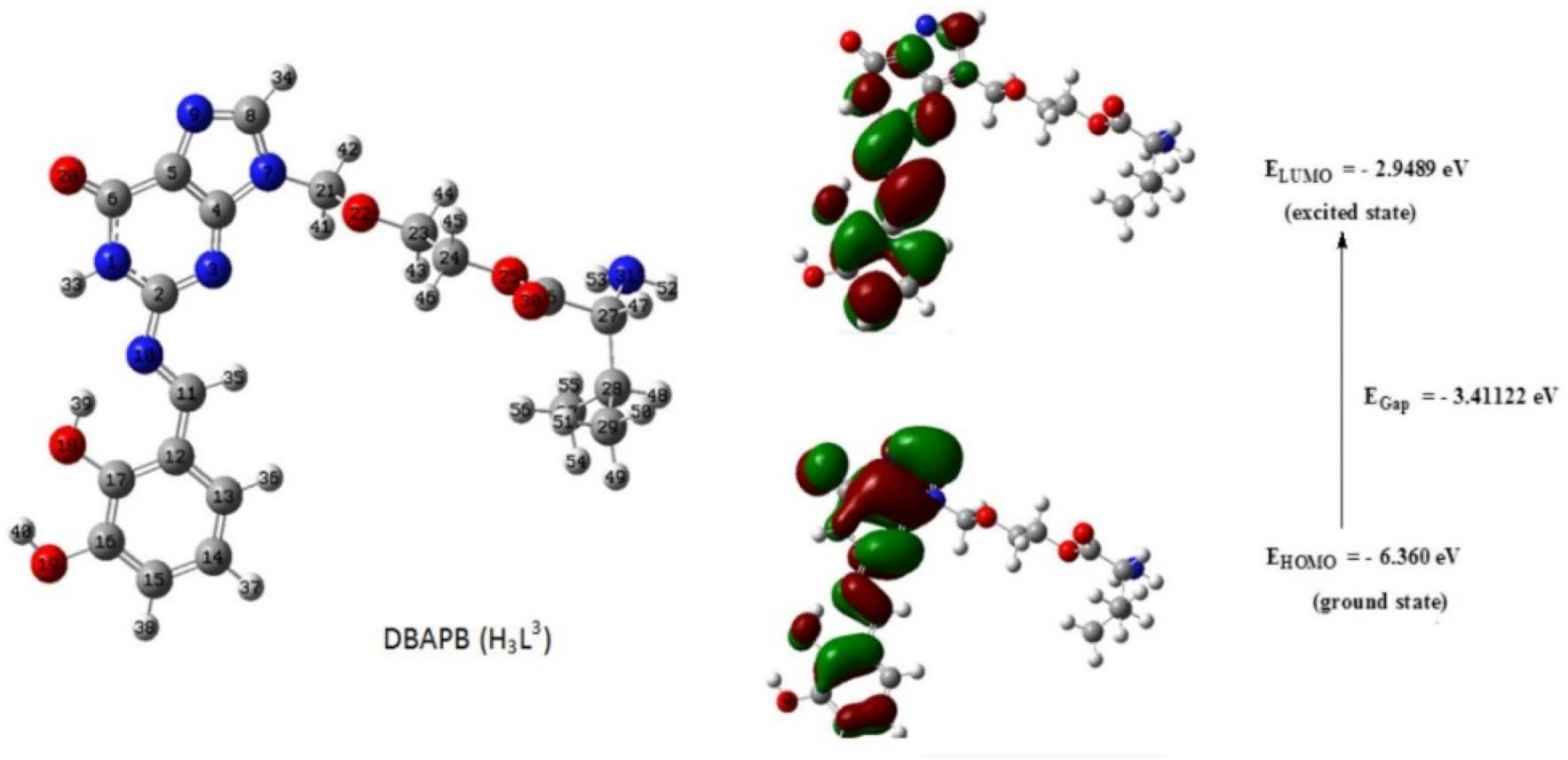
The optimized geometry and HOMO–LUMO energies for the ligand DBAPB.

**Figure 9:**
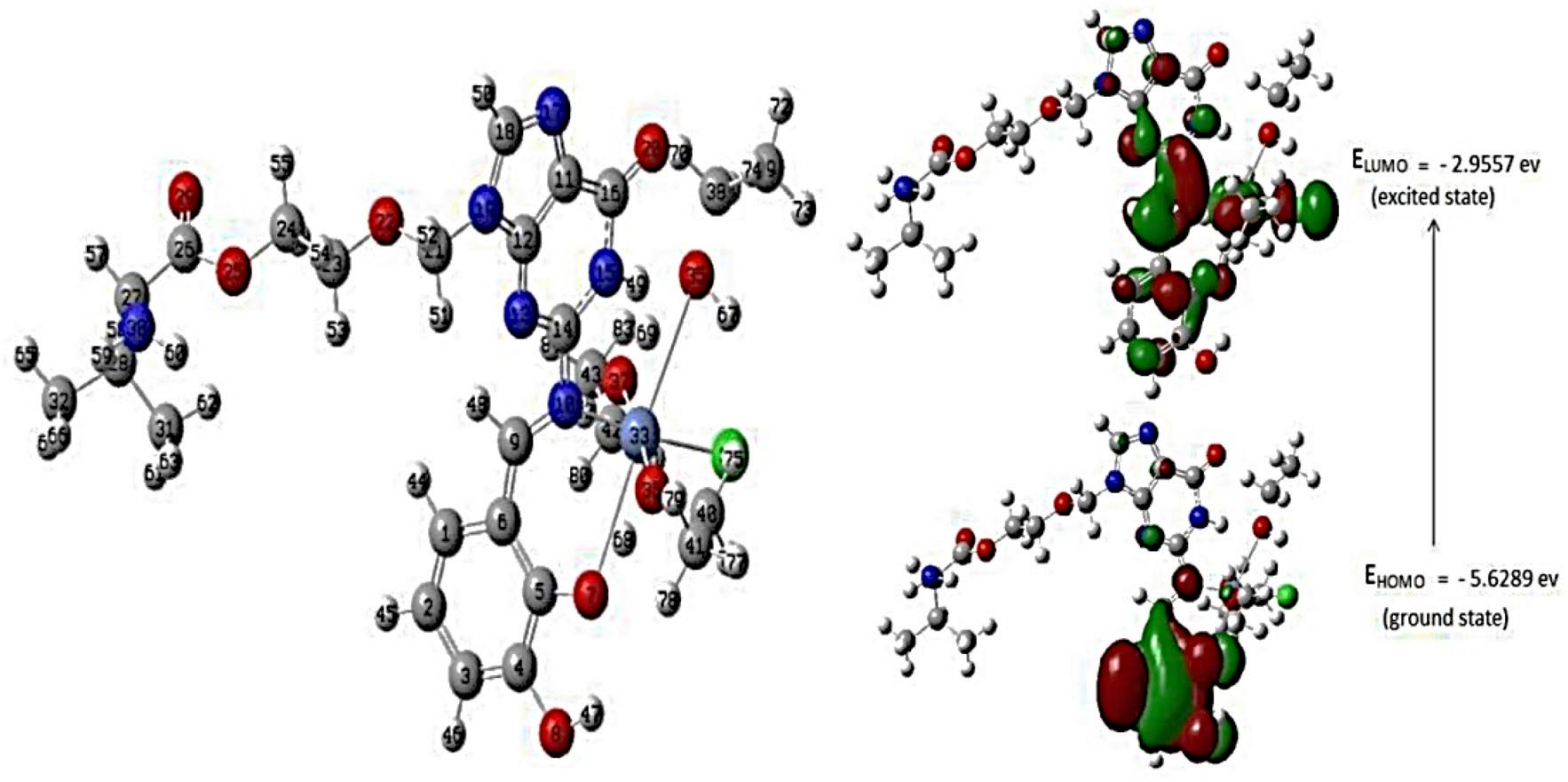
The optimized geometry and HOMO–LUMO energies for the Ni(II) complex 4.

**Figure 10:**
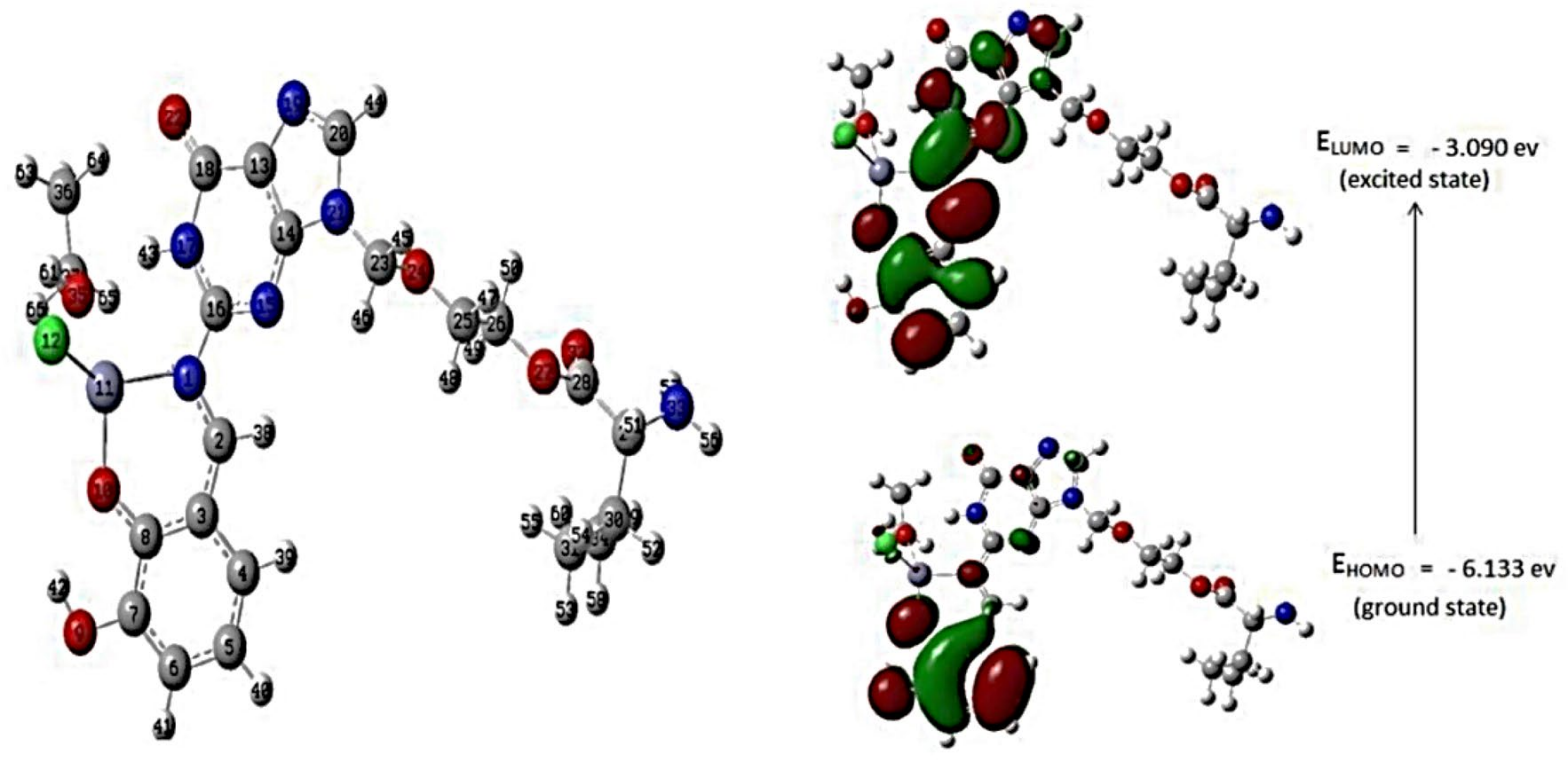
The optimized geometry and HOMO–LUMO energies for the Zn(II) complex 6.

The structures of the ligand, DBAPB, and its Cr(III), Mn(II), Co(II), Ni(II), Cu(II), Zn(II) complexes **1**–**6**, respectively, were optimized. The optimized molecular structures of the ligand, DBAPB, its Ni(II) and Zn(II) complexes **4** and **6**, respectively, were shown in Figs. 8, 9 and 10. The bond lengths and bond angles were evaluated and listed in Tables 5 and 6. The data showed that the bond lengths in the ligand, DBAPB, were somewhat changed upon complexation, particularly for the coordinated azomethine-N, C(11)-N(18) = 1.295 Å, and phenolate-O C(17)-O(18) = 1.348 Å, atoms. The computed azomethine (C=N) and phenolate (C–O) bond lengths in the complexes were in the ranges 1.284–1.371 and 1.323–1.356 Å, respectively, revealing slightly elongation of these bonds due to coordination with the metal ions. The M–N and M–O bond lengths were found to be in the range 1.948–2.198 and 1.852–2.199 Å, respectively. Also, the bond angles of the ligand were changed due to coordination. A greater change occurs for the angles that involving the coordinated azomethine N- and phenolate O- atoms, demonstrating their bonding with the metal ions. The estimated bond angles around the metal ions were found to be in the ranges of 76.103–171.099, 85.769–173.087, 78.7234–175.299 and 79.025–176.931° for Cl–M–O, Cl–M–N, O–M–O and N–M–O, angles (in all complexes except complex **6**), respectively, in addition Cl–Cr–Cl angle was found to be 171.477° (in the complex **1**). The values of bond angles indicated the octahedral configuration around the metal ions with d^2^sp^3^ or sp^3^d^2^ hybrid orbitals^46^. In contrast, the assessed bond angles around Zn(II) ion in the complex **6** were 87.195–125.806, 126.280, 121.355, and 93.815–102.508° for O-Zn-Cl, N-Zn-Cl, O–Zn–O and O-Zn-N angles, respectively. This suggests the tetrahedral geometry around Zn(II) ion^47^, which are consistent with the obtained experimental results.

**Table 5:** Evaluated bond lengths of the ligand, DBAPB, and its metal complexes.

Compound/complex	Bond	Length (Å)
DBAPB	C(11)-N(18)	1.295
	C(2)-N(18)	1.400
	C(17)-O(18)	1.348
	C(16)-O(19)	1.375
1	Cr(11)-O(37)	1.985
	Cr(11)-O(36)	1.920
	Cr(11)-Cl(35)	2.512
	Cr(11)-Cl(34)	2.437
	N(10)-Cr(11)	2.198
	O(7)-Cr(11)	1.852
2	Mn(33)-O(37)	2.188
	Mn(33)-O(36)	2.186
	Mn(33)-O(35)	2.199
	Mn(33)-Cl(34)	2.530
	N(10)-Mn(33)	2.170
	O(7)-Mn(33)	2.179
3	Co(33)-O(39)	2.136
	Co(33)-O(37)	2.067
	Co(33)-O(35)	1.930
	Co(33)-Cl(34)	2.647
	N(10)-Co(33)	1.985
	O(7)-Co(33)	1.873
4	N(10)-Ni(33)	1.948
	O(7)-Ni(33)	2.170
	O(35)-Ni(33)	2.181
	O(36)-Ni(33)	1.880
	O(37)-Ni(33)	1.921
	Cl(34)-Ni(33)	2.262
5	N(10)-Cu(33)	1.987
	O(7)-Cu(33)	1.954
	O(34)-Cu(33)	1.929
	O(35)-Cu(33)	2.064
	O(37)-Cu(33)	2.153
	Cl(36)-Cu(33)	2.537
6	N(1)-Zn(11)	2.084
	O(10)-Zn(11)	1.937
	O(35)-Zn(11)	2.126
	Cl(12)-Zn(11)	2.301

**Table 6:** Evaluated bond angles of the ligand, DBAPB, and its metal complexes.

Compound/complex	Angle	Degree (°)	Angle	Degree (°)
DBAPB	O(18)-C(17)-C(16)	117.897	H(35)-C(11)-N(18)	120.193
	O(18)-C(17)-C(12)	122.838	C(12)-C(11)-N(18)	122.309
	O(19)-C(16)-C(17)	120.947	C(11)-N(18)-C(2)	118.544
	O(19)-C(16)-C(15)	119.206	N(18)-C(2)-N(3)	123.144
1	O(37)-Cr(11)-O(36)	95.255	O(36)-Cr(11)-O(7)	101.394
	O(37)-Cr(11)-Cl(35)	77.647	Cl(35)-Cr(11)-Cl(34)	171.477
	O(37)-Cr(11)-Cl(34)	96.775	Cl(35)-Cr(11)-N(10)	99.700
	O(37)-Cr(11)-N(10)	82.222	Cl(35)-Cr(11)-O(7)	86.831
	O(37)-Cr(11)-O(7)	155.625	Cl(34)-Cr(11)-N(10)	85.769
	O(36)-Cr(11)-Cl(35)	82.856	Cl(34)-Cr(11)-O(7)	100.474
	O(36)-Cr(11)-Cl(34)	91.332	N(10)-Cr(11)-O(7)	82.017
	O(36)-Cr(11)-N(10)	175.894		
2	O(37)-Mn(33)-O(36)	85.062	O(36)-Mn(33)-O(7)	81.294
	O(37)-Mn(33)-O(35)	81.083	O(35)-Mn(33)-Cl(34)	88.389
	O(37)-Mn(33)-Cl(34)	163.987	O(35)-Mn(33)-N(10)	97.752
	O(37)-Mn(33)-N(10)	91.566	O(35)-Mn(33)-O(7)	179.428
	O(37)-Mn(33)-O(7)	99.067	Cl(34)-Mn(33)-N(10)	101.841
	O(36)-Mn(33)-O(35)	98.175	Cl(34)-Mn(33)-O(7)	91.347
	O(36)-Mn(33)-Cl(34)	84.531	N(10)-Mn(33)-O(7)	82.798
	O(36)-Mn(33)-N(10)	162.997		
3	O(39)-Co(33)-O(37)	87.790	O(37)-Co(33)-O(7)	82.044
	O(39)-Co(33)-O(35)	95.836	O(35)-Co(33)-Cl(34)	76.103
	O(39)-Co(33)-Cl(34)	171.099	O(35)-Co(33)-N(10)	96.782
	O(39)-Co(33)-N(10)	90.758	O(35)-Co(33)-O(7)	164.705
	O(39)-Co(33)-O(7)	93.284	Cl(34)-Co(33)-N(10)	93.911
	O(37)-Co(33)-O(35)	86.059	Cl(34)-Co(33)-O(7)	93.820
	O(37)-Co(33)-Cl(34)	87.890	O(37)-Co(33)-N(10)	176.931
4	O(7)-Ni(33)-N(10)	79.025	Cl(34)-Ni(33)-O(36)	94.9747
	O(7)-Ni(33)-Cl(34)	93.327	Cl(34)-Ni(33)-O(37)	90.0965
	O(7)-Ni(33)-O(36)	94.134	O(35)-Ni(33)-O(36)	131.0844
	O(7)-Ni(33)-O(37)	175.299	O(35)-Ni(33)-O(37)	78.7234
	N(10)-Ni(33)-O(35)	96.564	N(10)-Ni(33)-Cl(34)	173.087
	N(10)-Ni(33)-O(36)	165.251	Cl(34)-Ni(33)-O(35)	96.332
	N(10)-Ni(33)-O(37)	84.668		
5	O (7)-Cu(33)-N(10)	95.055	O(7)-Cu(33)-O(37)	80.729
	O(7)-Cu(33)-O(34)	163.051	N(10)-Cu(33)-O(35)	97.721
	O(7)-Cu(33)-O(35)	134.238	O(35)-Cu(33)-O(37)	91.047
	N(1)-Zn(11)-O(10)	93.815	O(10)-Zn(11)-Cl(12)	125.806
6	N(1)-Zn(11)-Cl(12)	126.280	O(10)-Zn(11)-O(35)	121.355
	N(1)-Zn(11)-O(35)	102.508	Cl(12)-Zn(11)-O(35)	87.195

### Frontier molecular orbitals HOMO–LUMO

The HOMO and LUMO values of the ligand, DBAPB, and its complexes **1**–**6**, were extracted from 3D plots of HOMO and LUMO, Figs. 8, 9 and 10, and the (HOMO–LUMO) gap energies (∆E) were calculated and listed in Table 7. The HOMO–LUMO energy gap explains the concluding charge transfer interaction within the molecule and is useful in determining molecular electrical transport properties. A molecule with a high frontier orbital gap (HOMO–LUMO energy gap) has low chemical reactivity and high kinetic stability because it is energetically unfavorable to add an electron to the high-lying LUMO in order to remove electrons from the low-lying HOMO. The compounds that have a high HOMO–LUMO energy gap are stable, and hence are chemically harder than compounds having a small HOMO–LUMO energy gap^48^. The sequence of the exact energy gap values of the ligand, DBAPB, and its complexes is as follows: Complex **2** < Complex **4** < Complex **1** < Complex **6** < Complex **5** < DBAPB < Complex **3**, which demonstrates that all metal complexes (except complex **3**) are more reactive than the free ligand, DBAPB, and the Mn(II) complex **2** exhibits the highest chemical reactivity in comparison to other complexes^48^. Also, the chemical reactivity values of the ligand, DBAPB, and its complexes: electronegativity (χ), chemical hardness (η), chemical potential (μ), electrophilicity (ω), and softness (S) were calculated and tabulated in Table 7. It was reported that a molecule is thought to be softer and more chemically reactive when its energy gap is small^48^. The softness (S) value of Mn(II) complex **2** was higher than that of the free ligand, DBAPB, and other complexes, which confirmed the higher chemical reactivity of this complex. Moreover, the values of the dipole moments were evaluated and shown in Table 7. The computed dipole moment (D) for the free ligand, DBAPB, and its complexes taking the following sequence: Complex **3** > Complex **2** > Complex **5** > DBAPB > Complex **6** > Complex **1** > Complex **4**, revealing that the Co(II) complex **3** has the highest polarity. According to the results of computational molecular properties, the examined metal complexes may therefore exhibit high bio-efficiency due to their high chemical reactivity than the free ligands, which is consistent with the reported experimental data.

**Table 7:** Evaluated quantum chemical parameters of the ligand, DBAPB, and its metal complexes.

Compound/complex	E_HOMO_ (ev)	E_LUMO_ (ev)	∆E (ev)	χ (ev)	μ (ev)	η (ev)	σ (ev)	S (ev)	ω (ev)	Dipole moment (D)
DBAPB	− 6.3601	− 2.9488	3.4112	4.6545	− 4.6545	1.7056	0.5863	0.2931	6.3509	9.65
1	− 6.0173	− 3.2058	2.8115	4.6116	− 4.6116	1.4057	0.7113	0.3556	7.5645	6.49
2	− 5.2480	− 2.7135	2.5345	3.9807	− 3.9807	1.2672	0.7891	0.3945	6.2523	11.29
3	− 6.724	− 3.0953	3.6287	1.6204	− 1.6204	1.8144	1.5302	0.2756	6.6428	14.14
4	− 5.6289	− 2.9557	2.6732	4.2923	− 4.2923	1.3366	0.7482	0.3741	6.8920	3.94
5	− 6.2248	− 3.0115	3.2134	4.6182	− 4.6182	1.6067	0.6223	0.3112	6.6371	11.00
6	− 6.1339	− 3.0909	3.0431	4.6125	− 4.6125	1.5215	0.6572	0.3286	6.9913	7.58

## Biological activity studies

### Antimicrobial activity

By using the disc diffusion method, the antimicrobial activity of the ligand, DBAPB, and its complexes was examined in vitro against a variety of pathogenic bacteria and fungi including *Escherichia coli, Staphylococcus aureus and Candida albicans*, and compared to the well-known standard medications: chloramphenicol (40 mm), cefoxitin (15 mm), and fluconazole (30 mm), respectively. The results obtained, Table 8, showed that the ligand, DBAPB, is inactive against all tested species except for *E. coli* with an inhibition zone 20 mm. The majority of the tested metal complexes showed potent antibacterial activity against both *E. coli* and *S. aureus*. The higher activity toward *S. aureus* appeared by complexes Co(II) and Cu(II), **15** and **23**, with inhibition zone values 29 and 30 mm, respectively, and higher than the standard drug cefoxitin (15 mm), revealing the high efficiencies of these complexes as antibacterial agents against *S. aureus*. The higher efficiency against *E. coli* was shown in the complexes Ni(II) and Zn(II), **20** and **29**, with inhibition zone values 30 and 25 mm, respectively. None of the metal complexes under investigation demonstrated antifungal activity against *C. albicans* with the exception of the complexes Cr(III), Mn(II) and Ni(II) **7**, **11** and **19**, which exhibited high activities with inhibition zone values 20, 20 and 25 mm, respectively. The antimicrobial activity results suggested that there is enhancement in activity of the free ligands upon coordination with the metal ions which can be explained based on chelation theory. Liposolubility is an important factor that controls the antimicrobial activity. On chelation the polarity of the metal ion will be reduced due to overlap of ligand orbital and partial sharing of the positive charge of the metal ion with donor groups. The increasing of delocalization of π-electrons over the whole chelate ring, resulting in an increase in the lipophilicity of the metal complexes. This improved lipophilicity enhances the concentration of complexes in the lipid membrane and limits the multiplicity of microorganisms. It is suggested that the antimicrobial activity of the complexes is due to either by killing the microbes or inhibiting their multiplication by blocking their active sites^49^.

**Table 8:** In vitro antimicrobial evaluation of the ligand, DBAPB, and its metal complexes.

Compounds	Mean* of zone diameter, nearest whole mm		
	Gram-positive bacteria	Gram-negative bacteria	Yeasts and Fungi**
	*Staphylococcus aureus* (ATCC 25923)	*Escherichia coli* (ATCC 25922)	*Candida albicans* (ATCC 10231)
DBAPB	–	20	–
1	–	12	–
2	–	–	–
3	–	15	–
4	–	12	–
5	–	9	–
6	9	9	–
7	–	20	20
8	–	24	–
9	–	15	–
11	24	20	20
12	–	19	–
13	–	–	–
15	29	17	–
16	–	–	–
17	–	–	–
19	27	22	25
20	20	30	–
21	20	15	–
23	30	16	–
24	–	–	–
25	–	–	–
29	15	25	–
30	–	20	–
31	10	20	–
# Control	15	40	30

*Calculate from 3 values.

**Identified based on routine cultural, morphological and microscopical characteristics. – = No effect.

^#^Cefoxitin in the case of Gram-positive bacteria, chloramphenicol in the case of Gram-negative bacteria and fluconazole in the case of fungi.

### Antitumor activity

**Figure 11:**
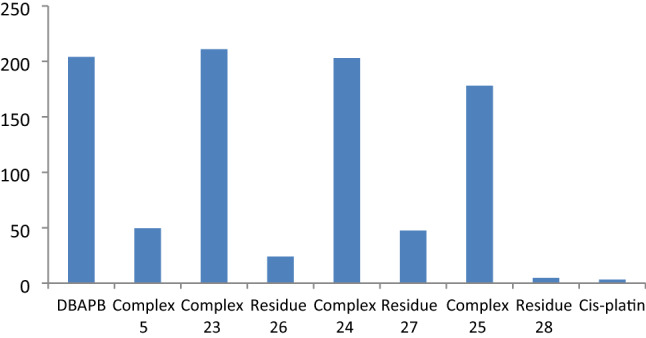
In vitro antitumor activity (IC_50_) of the ligand, DBAPB, and its Cu(II) complexes and their restudies against HepG-2 cells.

To study the antitumor effect, the ligand, DBAPB, and its Cu(II) complex **5** prepared in EtOH, in addition of its Cu(II) complexes **23**, **24**, **25** prepared in CTAB/EtOH, SO/EtOH, MP/EtOH, respectively, and their residues **26**, **27**, **28** produced after heating at 200 °C for 1 h, have been tested against Hepatocellular carcinoma cell line (HepG-2 cells). The common drug cis-platin was utilized for comparison (IC_50_ = 3.27 μg mL^−1^ against HepG-2 cell line). The IC_50_ value, which refers to the compound concentration that suppresses tumor cell growth by 50%, was used to specify the antitumor activity. Strong, moderate, and weak antitumor agents are classified as compounds with IC_50_ values less than 5.00, in the range of 5.00–10.00, and in the range of 10.00–25.00 μg mL^−1^, respectively^50^. The data obtained revealed that all screened compounds displayed an inhibition of cell viability and their IC_50_ (μg mL^−1^) values demonstrated in Fig. 11. While the ligand, DBAPB, and other tested complexes **5**, **23**, **24**, **25**, **26**, and **27** showed weak antitumor activities, with IC_50_ values in the range 24–211 μg mL^−1^ against HepG-2 cells., the residue **28**, which produced after heating the complex **25** at 200 °C for 1 h, exhibited strong antitumor activity with IC_50_ value 4.85 μg mL^−1^. The enhancement in antitumor activity after thermal treatment for nano-scale Cu(II) complex **25** may be explained due to the decrease in particle size of this complex under influence of heating, which facilitates the penetration of Cu(II) complex particles into the tumor cell and inhibits its growth. The highest cytotoxic activity of nano Cu(II) complex may be due to the high affinity of Cu(II) ions to bind DNA than any other divalent cation, thus promoting DNA oxidation^50^.

### DNA cleavage study

**Figure 12:**
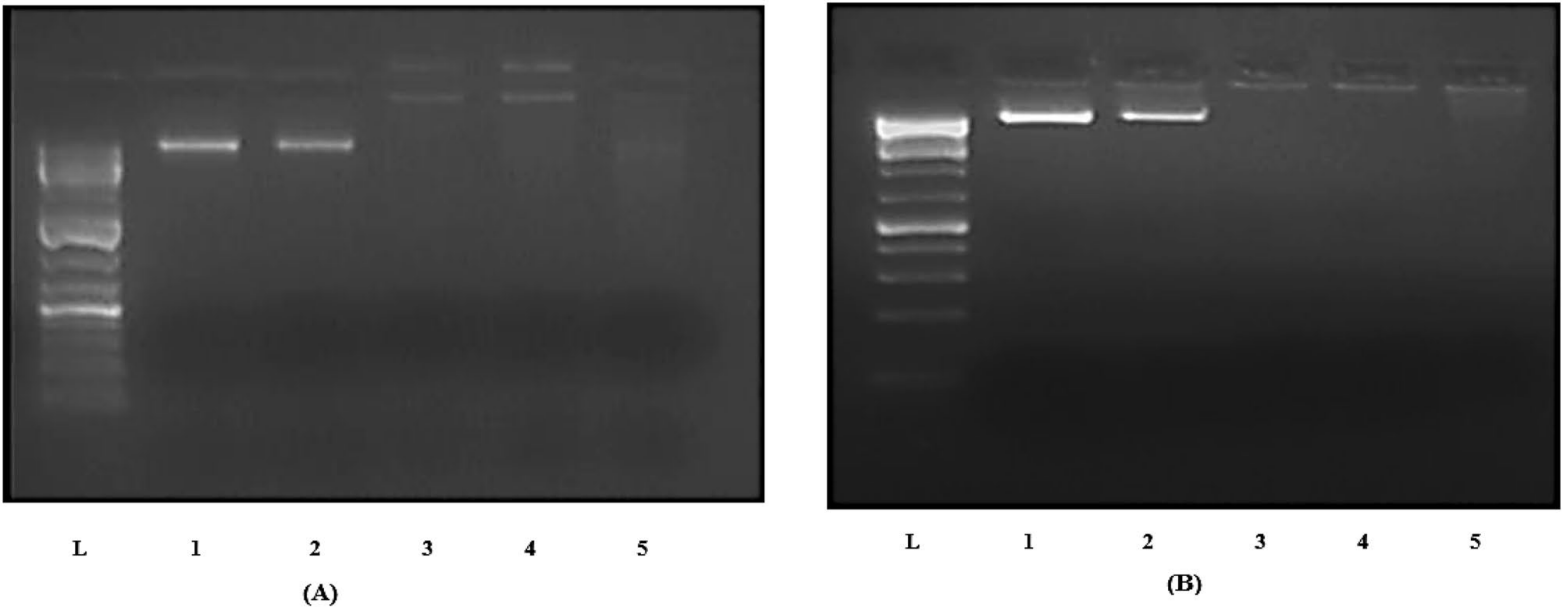
Electrophoretic separation of DNA induced by Cu(II) complex 24. (**A**) Fixed concentration of DNA with different concentrations of the complex (lane L- marker 1 kb DNA Ladder, lane 1: DNA control, lane 2: DNA + DMSO, lane 3: 400 ng DNA + 2 mg mL^−1^ of the complex, lane 4: 400 ng DNA + 1 mg ml^−1^ of the complex, lane 5: 400 ng DNA + 0.5 mg mL^−1^ of the complex). (**B**) Fixed concentration of complex with different concentrations of DNA (lane L-marker 1 kb DNA Ladder, lane 1: DNA control, lane 2: DNA + DMSO, lane 3: 200 ng DNA + 1 mg mL^−1^ of the complex, lane 4: 400 ng DNA + 1 mg mL^−1^ of the complex, lane 5: 800 ng DNA + 1 mg mL^−1^ of the complex).

Two experimental procedures-fixed DNA concentration with various complex concentrations and fixed complex concentration with various DNA concentrations- were used to examine the ability of Cu(II) complex **24** to cleave DNA. Figure 12 depicts the electrophoretic separation of DNA induced by the complex **24**. The findings demonstrated the ability of examined Cu(II) complex **24**, at the concentration of 1 mg mL^−1^, to degrade DNA at the concentration of 800 ng, indicating that this complex may function as antitumor agent and prevent the growth of tumor cells, because of its DNA-binding capacity.

### Molecular docking

**Figure 13:**
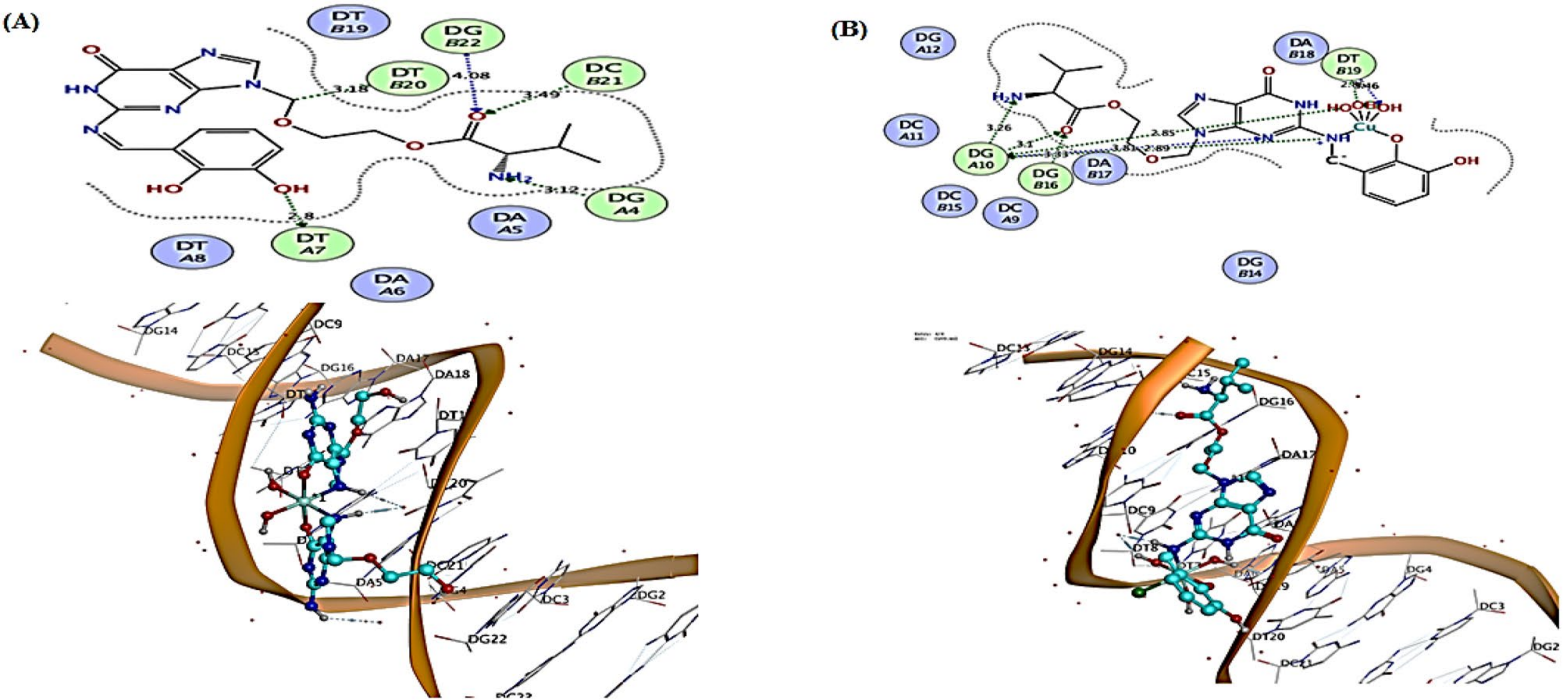
2D & 3D diagrams showing interaction between the ligand, DBAPB, (**A**), and its Cu(II) complex 5 (**B**) with DNA by molecular docking.

Molecular Operating Environment (MOE, 2015.10) software^22^ was used to conduct a computational molecular docking analysis of the ligand, DBAPB, and its Cu(II) complex **5** using DNA duplex of the dodecamer sequence (PDB ID: 1BNA). All minimizations were carried out with MOE until an RMSD gradient of 0.05 kcal mol^−1^ Å^−1^ with MMFF94x force field and the partial charges were automatically estimated. The synthetic DNA dodecamer d(CpGpCpGpApApTpTpCpGpCpG) crystal structure has been refined to 1.9-A resolution. The molecule forms slightly more than one full turn of right- handed double-stranded B helix. The two ends of the helix overlap and interlock minor grooves with nearby molecules up and down, producing a 19° bend in helix axis over the 11-base-pair steps of the dodecamer^51^. The data obtained, Table 9, showed that the ligand, DBAPB, and its Cu(II) complex **5** interact with DNA helix at the nucleotides DG-A4, DT-A7, DT-B20, DC-B21, DG-B22 and DG-A10, DG-B16, DT-B19 with binding energies (docking scores) of − 7.9549 and − 6.8852 kcal mol^−1^, respectively. The interacting groups with DNA were NH_2_, OH(phenolic), CH_2_, O=C–O, and NH_2_, O=C–O, azomethine (C=N), H_2_O for the ligand, H_3_L, and its Cu(II) complex **5**, respectively, by hydrogen bonding, as shown in Fig. 13. The docking results demonstrated strong interactions of both the ligand, DBAPB, and its Cu(II) complex **5**, revealing their capacity to cleave DNA and their potent inhibitory effects on tumor cells.

**Table 9:** Results of docking interactions to DNA of the ligand, DBAPB, and its Cu(II) complex 5.

Compound	Docking score S (kcal/mol)	DNA Base	Interacting groups	Type of interaction	H-bond length (Å)
Ligand, DBAPB	− 7.9549	DG-A4 DT-A7 DT-B20 DC-B21 DG-B22	NH_2_ OH (phenolic) CH_2_ O = C-O O = C-O	H-bond (acceptor) H-bond (donor) H-bond (donor) H-bond (acceptor) H-bond (acceptor)	3.12 2.80 3.18 3.49 4.08
Cu(II) complex 5	− 6.8852	DG-A10 DG-A10 DG-A10 DG-A10 DG-A10 DG-B16 DT-B19 DT-B19	NH_2_ O = C-O N azomethine (C = N) H_2_O O = C-O H_2_O H_2_O	H-bond (acceptor) H-bond (acceptor) H-bond (acceptor) H-bond (donor) H-bond (donor) H-bond (acceptor) H-bond (donor) H-bond (acceptor)	3.26 3.10 3.81 2.89 2.85 3.33 2.86 3.46

## Electrical conductivity measurements

**Figure 14:**
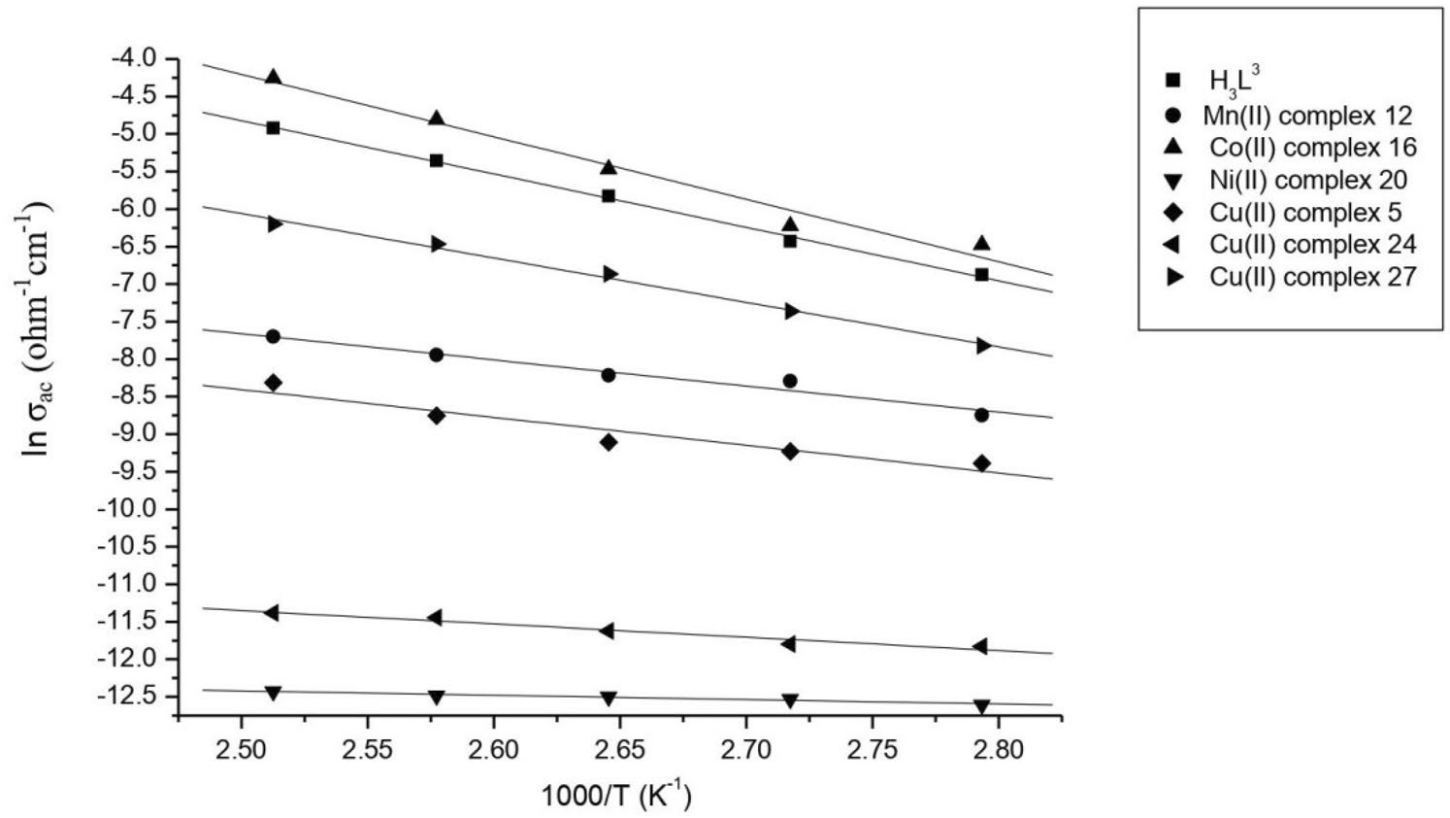
Temperature dependence of σ_ac_ of the ligand, DBAPB, and its complexes 5, 12, 16, 20, 24, 27.

The electrical conductivity (σ_ac_) of the ligand, DBAPB, and its solid complexes was measured as a function of temperature (T) over the range of 308–408 K, and a frequency range 0.1 kHz- 8 MHz using thin-film samples. Table 10 showed that as the temperature increases, the σ_ac_ of the ligand, DBAPB, and its Cu(II) complexes **5**, **24**, **27**, as well as its Mn(II), Co(II), and Ni(II) complexes, **12**, **16**, and **20**, respectively, increases. This suggests that the conduction process is thermally activated. In fact, several models were suggested to explain the conduction mechanisms in the materials of semiconductive behavior. For the compounds under consideration, the temperature dependence of the electrical conductivity is good described by the Arrhenius model^52^. This model assumes that the conduction mechanism is thermally activated, and it represents the relationship between electrical conductivity and temperature, shown in Fig. 14, by the equation: σ = σ^o^exp(− E_a_/k_B_T), where σ^o^, E_a_, and k_B_ are the pre-exponential factor, the activation energy for electrical conduction, and the Boltzmann's constant, respectively. The observed linear relationship between lnσ_ac_ and 1/T showed that there was an increase in σ_ac_ with temperature, which indicated that the compounds under investigation act as semiconductors. The mobility (μ) values, which are given by the formula: μ = σ/ne, (where e is the electron charge), and the charge carrier concentration (n) values, which given by the formula: n = 2(2πm*k_B_T/h^2^)^3/2^exp(− E_a_/k_B_T), (where m* is the effective mass of the charge carrier and assumed to be the rest mass of electron), were calculated and listed in Table 11 in order to characterize the mechanism of the conduction process. The compounds under study had mobility (μ) values in the 10^–23^–10^–37^ cm^2^ V^−1^ s^−1^ range, which are less than 1 cm^2^ V^−1^ s^−1^, indicated that the conduction process occurs through a hopping mechanism. The temperature dependence of the charge carrier concentration (n) in the low and high temperature ranges, Table 12, revealed that as the temperature increases, more charge carriers participate to the conduction process, confirming that the conduction process is of the thermally activated type.

**Table 10:** Variation of AC conductivity (σ_ac_) of the ligand, DBAPB, and its complexes 5, 12, 16, 20, 24, 27 with temperature, at frequency = 10 kHz.

T (k)	σ_ac_ (ohm^−1^ cm^−1^)						
	DBAPB	Mn(II) complex 12	Co(II) complex 16	Ni(II) complex 20	Cu(II) complex 5	Cu(II) complex 24	Cu(II) complex 27
308	3.14 × 10^–5^	1.17 × 10^–5^	2.78 × 10^–5^	3.31 × 10^–6^	2.64 × 10^–5^	2.00 × 10^–5^	7.29 × 10^–6^
318	3.56 × 10^–4^	1.24 × 10^–5^	2.93 × 10^–5^	3.35 × 10^–6^	2.67 × 10^–5^	2.04 × 10^–5^	7.52 × 10^–6^
328	4.77 × 10^–4^	9.83 × 10^–5^	3.50 × 10^–4^	3.62 × 10^–6^	2.70 × 10^–5^	2.13 × 10^–5^	8.93 × 10^–6^
338	5.02 × 10^–4^	1.59 × 10^–4^	5.86 × 10^–4^	3.73 × 10^–6^	2.96 × 10^–5^	6.52 × 10^–4^	1.07 × 10^–5^
348	6.66 × 10^–4^	2.50 × 10^–4^	9.98 × 10^–4^	3.79 × 10^–6^	8.38 × 10^–5^	7.09 × 10^–4^	1.14 × 10^–5^
358	1.03 × 10^–3^	2.70 × 10^–4^	1.51 × 10^–3^	4.01 × 10^–6^	9.81 × 10^–5^	2.56 × 10–3	1.22 × 10^–5^
368	1.61 × 10^–3^	3.54 × 10^–4^	1.54 × 10^–3^	5.38 × 10^–6^	1.11 × 10^–4^	5.81 × 10–3	1.34 × 10^–5^
378	2.95 × 10^–3^	4.52 × 10^–4^	1.98 × 10^–3^	1.14 × 10^–5^	1.58 × 10^–4^	7.27 × 10–3	1.68 × 10^–5^
388	4.72 × 10^–3^	4.53 × 10^–4^	4.22 × 10^–3^	2.38 × 10^–5^	2.45 × 10^–4^	8.95 × 10–3	1.88 × 10^–5^
398	7.29 × 10^–3^	4.75 × 10^–4^	8.15 × 10^–3^	4.61 × 10^–4^	2.47 × 10^–4^	4.93 × 10^–2^	6.22 × 10^–5^
408	1.57 × 10^–2^	4.95 × 10^–3^	1.42 × 10^–2^	8.46 × 10^–4^	3.45 × 10^–4^	5.24 × 10^–2^	1.24 × 10^–4^

**Table 11:** Electrical properties of the ligand, DBAPB, and its complexes 5, 12, 16, 20, 24, 27 at 308 K.

Compound	σ_ac_ (ohm^−1^ cm^−1^)	E_a_(ev)	n (cm^−3^)	µ (cm^2^ V^−1^ s^−1^)
DBAPB	6.57 × 10^–5^	0.61	1.41 × 10^41^	2.92 × 10^–27^
Mn(II) complex 12	4.23 × 10^–5^	0.30	1.05 × 10^44^	2.52 × 10^–30^
Co(II) complex 16	4.23 × 10^–5^	0.72	1.84 × 10^37^	1.44 × 10^–23^
Ni(II) complex 20	2.70 × 10^–6^	0.05	1.12 × 10^50^	1.51 × 10^–37^
Cu(II) complex 5	1.68 × 10^–5^	0.32	5.02 × 10^43^	2.09 × 10^–28^
Cu(II) complex 24	3.91 × 10^–5^	0.51	4.43 × 10^40^	5.51 × 10^–25^
Cu(II) complex 27	3.80 × 10^–6^	0.15	2.71 × 10^46^	8.76 × 10^–32^

**Table 12:** Variation of charge carrier concentration (n) of the ligand, DBAPB, and its complexes 5, 12, 16, 20, 24, 27 with temperature.

Charge carrier concentration (n) cm^−3^							
T (k)	DBAPB	Mn(II) complex 12	Co(II) complex 16	Ni(II) complex 20	Cu(II) complex 5	Cu(II) complex 24	Cu(II) complex 27
308	1.41 × 10^41^	1.05 × 10^44^	1.84 × 10^37^	1.12 × 10^50^	5.02 × 10^43^	4.43 × 10^40^	2.71 × 10^46^
318	1.59 × 10^41^	1.29 × 10^46^	2.85 × 10^39^	1.18 × 10^50^	6.24 × 10^45^	6.12 × 10^42^	3.10 × 10^48^
328	3.09 × 10^41^	1.93 × 10^46^	6.91 × 10^39^	1.31 × 10^50^	9.43 × 10^45^	1.15 × 10^43^	3.86 × 10^48^
338	6.46 × 10^41^	2.69 × 10^46^	1.45 × 10^40^	1.45 × 10^50^	1.37 × 10^46^	1.97 × 10^43^	4.68 × 10^48^
348	1.17 × 10^42^	3.72 × 10^46^	2.94 × 10^40^	1.60 × 10^50^	1.90 × 10^46^	3.29 × 10^43^	5.57 × 10^48^
358	2.19 × 10^42^	5.19 × 10^46^	6.18 × 10^40^	1.76 × 10^50^	2.71 × 10^46^	5.67 × 10^43^	6.76 × 10^48^
368	4.04 × 10^42^	7.15 × 10^46^	9.18 × 10^40^	1.93 × 10^50^	3.81 × 10^46^	9.46 × 10^43^	8.11 × 10^48^
378	7.17 × 10^42^	9.66 × 10^46^	2.44 × 10^41^	2.10 × 10^50^	5.2 × 10^46^	1.54 × 10^44^	9.61 × 10^48^
388	1.16 × 10^43^	1.25 × 10^47^	3.98 × 10^41^	2.29 × 10^50^	6.87 × 10^46^	2.32 × 10^44^	1.12 × 10^49^
398	1.95 × 10^43^	1.64 × 10^47^	8.26 × 10^41^	2.37 × 10^50^	9.15 × 10^46^	3.59 × 10^44^	1.30 × 10^49^
408	3.20 × 10^43^	2.14 × 10^47^	1.39 × 10^42^	2.57 × 10^50^	1.21 × 10^47^	5.46 × 10^44^	1.51 × 10^49^

## Conclusion

**Figure 15:**
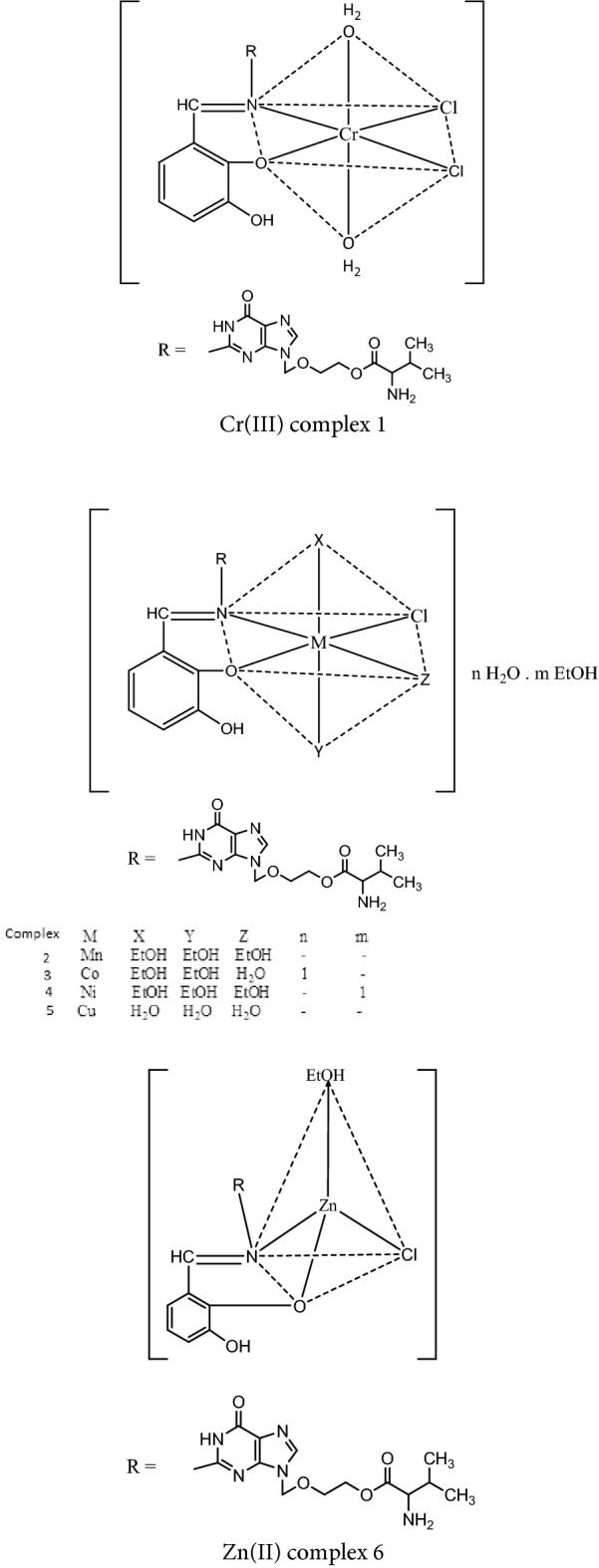
Suggested coordination of the investigated complexes 1–6.

[Cr(DBAPB)(H_2_O)_2_Cl_2_], [Mn(DBAPB)(EtOH)_3_Cl], [Co(DBAPB)(EtOH)_2_(H_2_O)Cl].H_2_O, [Ni(DBAPB)(EtOH)_3_Cl]. EtOH, [Cu(DBAPB)(H_2_O)_3_Cl] and [Zn(DBAPB)(EtOH)Cl] were synthesized by the reaction of Cr(III), Mn(II), Co(II), Ni(II), Cu(II) and Zn(II) with the Schiff base ligand, (2-{(2-[(2,3-dihydroxy-benzylidene)-amino]-6-oxo-1H-purine-9-yl)methoxy}ethyl-2-amino-3-mehylbutanoate), DBAPB. Thermal, magnetic, spectroscopic, and analytical methods were used to characterize all reported complexes. The ligand, DBAPB, behaves as bidentate towards the metal ions. Except for the Zn(II) complex, which had tetrahedral structure, all the examined complexes were suggested to have octahedral geometry. The findings of the thermal decomposition showed that the newly synthesized complexes have a high thermal stability. According to the XRD and TEM data, the particles of the examined complexes were situated in nano range. The data obtained from the theoretical study agreed with the experimental results. Biological activity of the ligand, DBAPB, and its complexes concluded that the metal ion in the complexes enhanced the antimicrobial activity compared to the free ligand. Additionally, all compounds under investigation showed an inhibition of HEPG-2 cell growth, and the antitumor activity of the complexes was improved after heating of them at 200 °C for 1 h. Moreover, the DNA cleavage investigation demonstrated the capacity of the examined Cu(II) complex to degrade DNA. The molecular docking study indicated that the ligand, DBAPB, and its investigated Cu(II) complex are capable of cleaving DNA. The electrical conductivity studies indicated the semiconducting character for the ligand, DBAPB, and its investigated complexes within the temperature range 308–408 K, and the conduction process is carried out via the hopping mechanism. The obtained results showed the suggested structures of the complexes under investigation, as demonstrated in Fig. 15.

## Data Availability

All data supporting the findings of this study are available within the article.
